# Akt3 links mitochondrial function to the regulation of Aurora B and mitotic fidelity

**DOI:** 10.1371/journal.pone.0315751

**Published:** 2025-03-06

**Authors:** Zachary J. Hough, Fatemeh Nasehi, Daniel G. Corum, Russell A. Norris, Ann C. Foley, Robin C. Muise-Helmericks

**Affiliations:** 1 Department of Regenerative Medicine and Cell Biology, The Medical University of South Carolina, Charleston, South Carolina, United States of America; 2 Department of Bioengineering, Clemson University, Clemson, South Carolina, United States of America; King Faisal Specialist Hospital and Research Center, SAUDI ARABIA

## Abstract

Akt3 is a key regulator of mitochondrial homeostasis in the endothelium. Akt3 depletion results in mitochondrial dysfunction, decreased mitochondrial biogenesis, and decreased angiogenesis. Here we link mitochondrial homeostasis with mitotic fidelity—depletion of Akt3 results in the missegregation of chromosomes as visualized by multinucleation and micronuclei formation. We have connected Akt3 to Aurora B, a significant player in chromosome segregation. Akt3 localizes to the nucleus, where it associates with and regulates WDR12. During mitosis, WDR12 is localized to the dividing chromosomes, and its depletion results in a similar mitotic phenotype to Akt3 depletion. WDR12 associates with Aurora B, both of which are downregulated under conditions of Akt3 depletion. We used the model oxidant paraquat to induce mitochondrial dysfunction to test whether the Akt3-dependent effect on mitochondrial homeostasis is linked to mitotic function. Paraquat treatment also causes chromosome missegregation by inhibiting the expression of Akt3, WDR12, and Aurora B. The inhibition of ROS rescued both the mitotic fidelity and the expression of Akt3 and Aurora B. Akt3 directly phosphorylates the major nuclear export protein CRM-1, causing an increase in its expression, resulting in the inhibition of PGC-1 nuclear localization, the master regulator of mitochondrial biogenesis. The Akt3/Aurora B pathway is also dependent on CRM-1. CRM-1 overexpression resulted in chromosome missegregation and downregulation of Aurora B similar to that of Akt3 depletion. Akt3 null hearts at midgestation (E14.5), a stage in which proliferation is occurring, have decreased Aurora B expression, increased CRM-1 expression, decreased proliferation, and increased apoptosis. Akt3 null hearts are smaller and have a thinner compact cell layer than age-matched wild-type mice. Akt3 null tissue has dysmorphic nuclear structures, suggesting mitotic catastrophe. Our findings show that mitochondrial dysfunction induced by paraquat or Akt3 depletion results in a CRM-1-dependent disruption of Aurora B and mitotic fidelity.

## Introduction

Endothelial cells (ECs) rely mainly on glycolysis for energetic requirements, having a relatively low mitochondrial content that does not contribute to cellular ATP levels in a meaningful way [1,2]. This contrasts with the high metabolic requirements of the heart and kidney, which require mitochondrial oxidative phosphorylation for ATP production. It is well-established that mitochondria perform functions independent of ATP generation, including the regulation of apoptosis, ROS production, calcium signaling, and iron handling. Mitochondrial homeostasis is controlled by a balance of mitochondrial biogenesis, autophagy, and fission/fusion processes that maintain mitochondrial health [3–5]. Increased mitochondrial biogenesis produces healthy mitochondria in times of increased energetic requirements and, for the dilution of damaged mitochondria, during times of stress. Fission and fusion events between mitochondria distribute undamaged mitochondrial components throughout the entire mitochondrial pool [6]. Mitochondrial dysfunction causes a significant increase in ROS production, which can overwhelm cellular antioxidant defenses contributing to the pathogenesis of disease states [7,8]. Interestingly, mitochondrial function is essential to EC homeostasis, with perturbations to mitochondrial homeostasis being attributed to various vascular disorders. Studies have found that mitochondria serve a critical function as sensors of extracellular signals, including hypoxia, shear stress, nutrients, and caloric restriction [7]. Disruptions in mitochondrial homeostatic processes have been implicated in the vasculature, resulting in decreased endothelial barrier function and angiogenesis [9]. These disruptions can lead to other pathologies, including cardiovascular and neurodegenerative diseases.

Our previous studies show that Akt3 is required to launch angiogenic responses [10]. Importantly, ECs recruited during an angiogenic challenge in Akt3 null mice have fewer mitochondria, a phenotype not shared with Akt1 null mice. Akt3 regulates mitochondrial biogenesis, dynamics, and localization, at least in part, by regulating the nuclear accumulation of PGC1α indirectly via control of CRM-1 (exportin 1) expression. Mitochondrial-nuclear communication is a well-established phenomenon linking mitochondrial function to nuclear gene expression, including those involved in the induction of mitosis. Here we show that EC mitochondrial dysfunction induced either by depletion of Akt3 or treatment with the model oxidant paraquat, results in inappropriate chromosome segregation, characterized by the formation of micronuclei and multinucleation. Accordingly, each of these treatments resulted in a decrease in the expression of the nuclear scaffolding protein WDR12, and the spindle assembly checkpoint protein Aurora B, which we have found to associate during mitosis. These processes are dependent on CRM-1. CRM-1 overexpression phenocopies the mitotic catastrophe induced by Akt3 depletion. Consistent with these findings, we observed that embryonic hearts of Akt3 null animals showed increased CRM-1 expression, decreased Aurora B expression, decreased proliferation, and increased cell death, leading to a heart defect that has not previously been described in Akt3 null mice. These findings link mitotic fidelity to mitochondrial homeostasis through a novel Akt3 signaling pathway. This pathway may provide a mechanism to explain how ROS leads to mitotic defects.

## Materials and methods

### Cell culture, transfection, and drug treatments

Pooled, multiple donor human umbilical vein cells (EC) (Lonza, Basal, Switzerland) were maintained at 37°C with 5% CO_2_ in endothelial basal medium 2 (EBM, Lonza) supplemented with EGM-2 SingleQuots. EC was transfected using an Amaxa Nucleofection system as described by the manufacturer. Briefly, 2 X 10^6^ cells were transfected per cuvette, using no more than 6µg of vector per transfection. The efficiencies of all transfections were monitored via GFP expression using a GFP expression vector pGFP-C1 (Clontech, Mountainview, CA) or a GFP-directed RNAi (Amaxa, Gaithersburg, MD). Smart Pool RNAi was purchased from Dharmacon (Akt3) or Santa Cruz Biotechnology (Akt3 and PGC-1α). EC were treated with paraquat (Sigma, final concentration 1mM) for the times indicated in the text. N-acetyl cysteine (NAC), MitoQ, and TAME were purchased from Cayman Chemicals. MitoSox was purchased from Invitrogen.

### Nuclear extract isolation and mass spectrometry

Nuclear extracts were isolated from HUVEC using a nuclear cytoplasmic fractionation kit (Pierce) and subjected to immunoprecipitation using antibodies directed against Akt1 or Akt3 and isolated using SDS-PAGE followed by Coomassie blue staining. Akt3 specific bands were subjected to peptide sequencing using a Thermo Orbitrap Elite with VelosPro Ion Trap MS by the MUSC Mass Spectrometry Core Facility directed by Lauren Ball, Ph.D.

### Real-time PCR analysis

cDNA was synthesized from 2 μg of total RNA with a Superscript First Strand Synthesis Kit purchased from Invitrogen, Inc, using Oligo(dT) according to the manufacturer’s instructions. Real-time PCR was performed using a Light Cycler 480, from Roche Diagnostics. For each experiment, n ≥  3 for each respective group and was performed at least in triplicate. Primer sequences are as follows (written 5’ to 3’). WDR12 Forward: CACGCTTCTACACTGATAACAAGAA, Reverse: GATGATGTTACTAAGGTCGGCAAT; S26 Forward: CTCCGGTCCGTGCCTCCAAG, Reverse: CAGAGAATAGCCTGTCTTCAG

### Immunoblot analysis

Antibodies used for Immunoblot analysis are Akt3 (Millipore), Akt1 (Santa Cruz Biotechnology), WDR12 (GeneTex), Aurora B, CRM-1, and Lamin A/C (Millipore), Actin (Abcam), Ki67 and cleaved caspase 3 (BD Pharmingen). Appropriate HRP-conjugated secondary antibodies were purchased from Invitrogen. Treated cells were washed once with PBS and lysed in 1X RIPA Lysis Buffer (50 mM Tris-HCl, pH 7.5, 1% Triton X100, 150 mM NaCl, 0.1% SDS, 1% sodium deoxycholate, 40 mM NaF), supplemented with complete protease inhibitors without EDTA (Roche, Palo Alto, CA) and 200 µ M sodium orthovanadate. Protein concentrations were measured using the BCA protein assay (Pierce, Rockford, IL), resolved by SDS-PAGE and transferred onto Immobilon-P PVDF membranes (Millipore). Immunoblots were visualized with luminol reagent (Santa Cruz).

### Veterinary care

AAALAC accreditation status of the Ralph H. Johnson VAMC Veterinary Medical Unit (VMU): Animals are housed in the facilities for laboratory animals provided by the VMU under the direction of M.A. McCrackin, DVM, PhD, a diplomate of both ACVS and ACLAM and an AALAS Certified Manager of Animal Resources. Dr. McCrackin is also a member of the 2013 AVMA Panel on Euthanasia. An Animal Welfare Assurance (A3137) is on file with the Office of Laboratory Animal Welfare (OLAW) detailing the program for laboratory animal care. The VMU has been fully accredited by AAALAC since 1974 and was most recently re-accredited in July 2014.

### Immunocyotochemistry

Antibodies used for immunofluorescence are described above except for α-tubulin (Sigma). Phalloidin was purchased from Molecular Probes, Invitrogen. All fluorescent-tagged secondary antibodies were purchased from Molecular Probes, Invitrogen. Transfected or treated ECs were seeded onto poly-L-lysine coated cover slips, fixed in 3.7% formaldehyde for 20 min and washed briefly in 1X PBS. Cells were permeabilized in 0.1% Triton 100-X for 20 min, washed briefly in 1X PBS, and blocked for 30 min in 5% BSA-PBS under gentle agitation. Cells were subsequently incubated in primary antibody for 1 hr at room temperature, and then washed in 1X PBS and incubated with the appropriate fluorescent secondary antibody for 1 hr at room temperature. After washing in 1X PBS, cell nuclei were stained with DAPI nuclear dye (Molecular Probes) and coverslips were mounted to slides using Fluorogel (Electron Microscopy Sciences, Hatfield, PA). Coverslips were imaged on a Zeiss Axio Imager M2 fluorescent microscope.

### Immunohistochemistry and microscopy

Paraffin embedded tissue sections were re-hydrated through xylene and a series of graded alcohols. Sections were submerged in antigen retrieval reagent (Vector Laboratories) and water in a pressure cooker for one minute and then washed with 1X PBS and blocked in 1% BSA-PBS for 30 minutes. Samples were incubated in Background Buster (Innovex Biosciences) for 30 minutes before antibody labeling. Sections were incubated in blocking buffer at 4°C overnight in primary antibody, washed, then incubated with an appropriate secondary antibody for 1 hour at room temperature. Cell nuclei were stained with DAPI nuclear dye (Invitrogen) then sections were mounted using KPL mounting Medium (Sera care). Primary antibodies included: rabbit anti-Aurora b (Millipore), rabbit anti-KI67 (BD Pharmingen), rabbit anti-cleaved caspase-3 polyclonal (1:200; BD Pharmingen). Secondary antibodies included: Alexa 488-conjugated anti-mouse IgG, Alexa 555-conjugated anti-rabbit IgG. Tissue sections were visualized using Zeiss Axio fluorescent microscope.

### Hematoxylin and Eosin Staining (H&E)

For H&E staining sections were cleared in xylene, rehydrated through a series of graded alcohols, placed in hematoxylin followed by acid alcohol. Samples were then placed in ammonia water, rinsed in ethanol, and exposed to eosin before dehydrating through graded alcohols and clearing in xylene. Sections were mounted using Cytoseal-XYL (Richard- Allan Scientific). H&E sections were visualized using an Olympus BX40 microscope and captured using an Olympus Camera (Model DP25).

### Statistics

All statistical analyses of mouse histological sections were performed using Microsoft Excel or Prism to calculate means and standard deviations. The results are presented as the mean ±  SEM values. For comparisons between two groups, a Student’s t-test was used. A value of p <  0.05 (*) was considered statistically significant and p < 0.01(**) statistically very significant.

## Results

### Akt3 depletion results in micronuclei formation in primary endothelial cells

**Fig 1:**
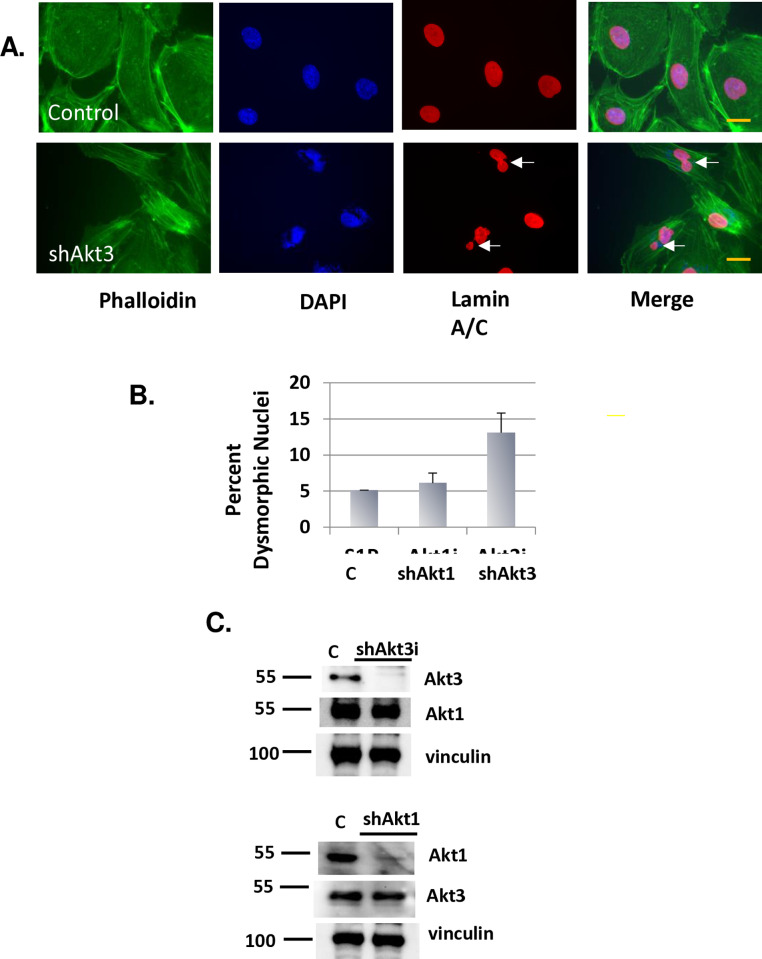
Akt3 depletion results in micronuclei formation. A) ECs were subjected to Akt3 depletion or scrambled control. Fixed cells were assessed for nuclear structure using immunofluorescence with antibodies directed against Lamin A/C and actin stained with phalloidin. DAPI is used as a nuclear stain. Arrows point to multinuclei and blebbing. 60X images are shown B) Quantitation of micronuclei (MN) under conditions of scrambled control, or Akt1 or Akt3 depletion. Standard deviation and p values are shown. C) Confirmation of Akt1 and Akt3 knockdown by immunoblot analysis. The PI3 kinase p85 subunit is shown as a loading control.

Our studies have shown that Akt3 is required for angiogenesis due to its control of mitochondrial biogenesis. During our studies we noticed that Akt3 depletion in ECs resulted in an accumulation of multinucleated cells and cells containing micronuclei, hallmarks of chromosome missegregation [11] (Fig 1A). Quantitation of this phenotype indicated that Akt3 depletion resulted in a 2.6-fold increase in micronuclei and multinucleation (Fig 1B). In addition, lamin A/C staining, which marks the nuclear envelope, shows nuclear blebbing under conditions of Akt3 depletion. Confirmation of the specificity of each RNAi is shown in Fig 1C. These findings suggest that Akt3, not Akt1, is required for appropriate chromosome segregation. Additional pictures of the mitotic and nuclear blebbing are shown in Supplemental Figures 1–2 (S3 File).

### WDR12 is required for appropriate chromosome segregation

Mass spectrophotometry was used to determine potential Akt3-nuclear protein interactions. Nuclear fractionation was performed and nuclear extracts were subjected to immunoprecipitation using antibodies directed against either Akt1 or Akt3. Protein bands specifically immunoprecipitated by Akt3 antibodies, were used for analysis by mass spectrophotometry. A list of potential protein partners is shown in Supplemental Figure 3 (S2 File). As anticipated several proteins that associate with Akt3 are involved in ribosome biogenesis [12]. We focused on WDR12, a nuclear scaffolding protein that regulates mitosis in plants and yeast [13–15] and is defined by GWAS as a player in vascular diseases [15–17].

**Fig 2:**
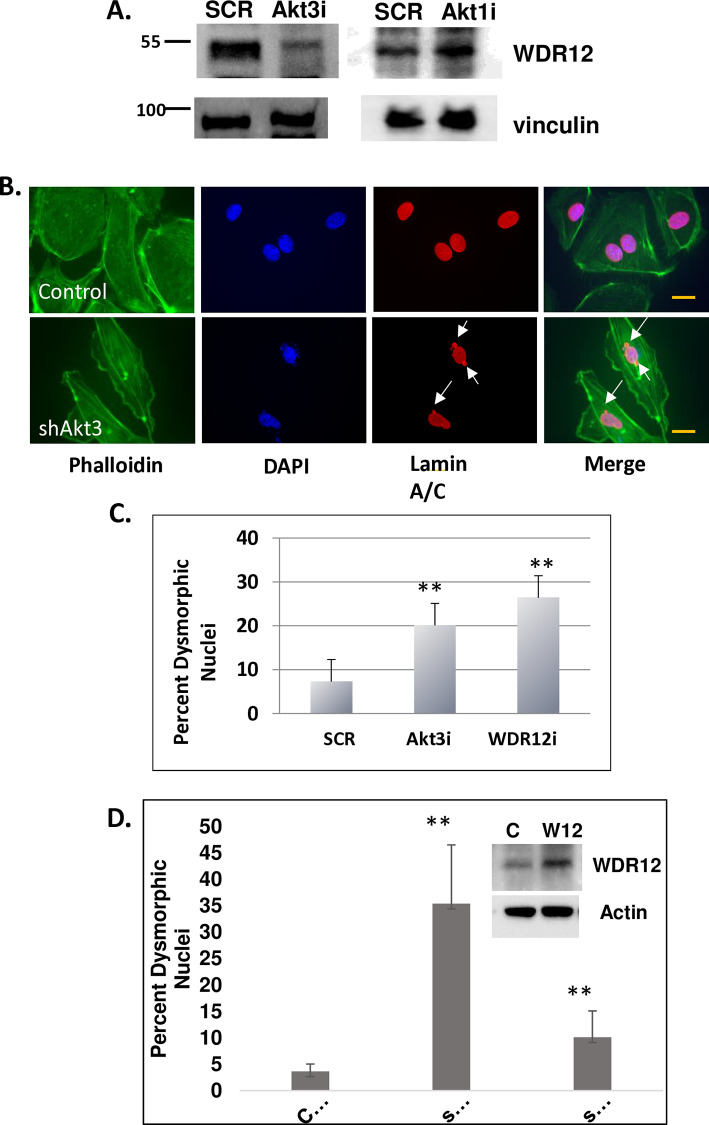
Akt3 controls the expression of WDR12, a chromosome binding protein. A) ECs were transfected with an RNAi directed against scrambled control or Akt3 and assessed for expression of WDR12 by Immunoblot analysis. p85 is shown as a loading control. B) Visualization of micronuclei (MN) in response to WDR12 depletion. C) Percent micronuclei was measured in ECs transfected with scrambled control, or RNAi directed against Akt3 or WDR12. Standard deviation is shown. * p < 0.05, **p < 0.05 D) Quantitation of dysmorphic nuclei resulting from overexpression of WDR12 in Akt3 depleted cells.

To test the effect of Akt3 on WDR12, we first used co-immunoprecipitation to determine if these proteins associate. Although we were unable to confirm a consistent and stable interaction between Akt3 and WDR12, Akt3 depletion resulted in a reduction in WDR12 protein expression (Fig 2A) which is not due to changes in WDR12 mRNA expression (data not shown). This suggests a post-transcriptional mechanism of Akt3 control of WDR12. To determine if WDR12 was also involved in the Akt3-dependent mitotic pathway, WDR12 was depleted using RNAi, and the effect on micronuclei formation was tested. As shown in Fig 2B, depletion of WDR12 results in a 5-fold increase in multinucleation and micronuclei formation (Fig 2C), similarly to that shown for Akt3 depletion. To confirm that WDR12 was indeed downstream of Akt3, Akt3 depleted cells were transfected with a WDR12 expression construct, cells were visualized by immunofluorescence, and dysmorphic nuclei quantitated. As shown by the quantitation in Fig 2D overexpression of WDR12 under conditions of Akt3 depletion rescues the dysmorphic nuclear phenotype. Collectively, these findings suggest that WDR12 plays a key role in mitosis, and is required for appropriate chromosome segregation.

### Akt3/WDR12 pathway impinges upon Aurora B kinase

**Fig 3:**
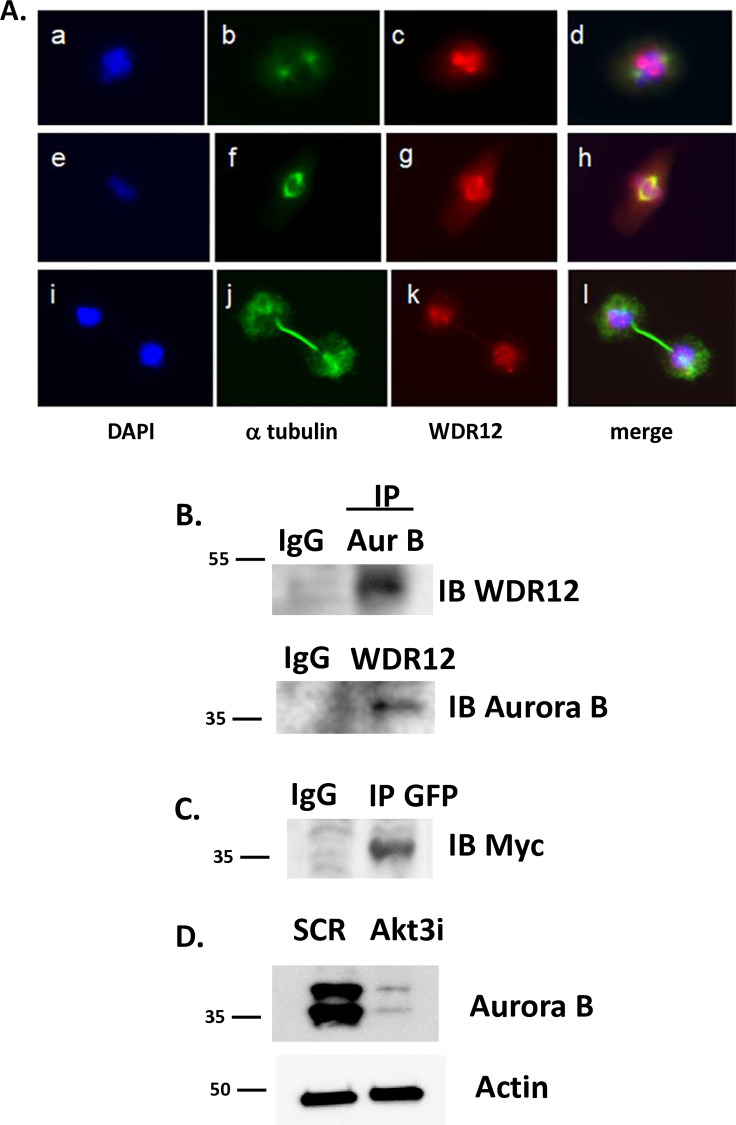
WDR12 associates with Aurora B. A) Immunofluorescence using antibodies against αtubulin, and WDR12 showing WDR12 localization during different stages of mitosis. DAPI is the nuclear stain. B) Immunoprecipitation of Aurora B or WDR12 followed by immunoblot of WDR12 or Aurora B, respectively. C) Co-immunoprecipitation ECs transfected with GFP-tagged Aurora B plus Myc-tagged WDR12 using an antibody directed against GFP followed by immunoblot with anti-Myc. D) RNAi directed against Akt3 or scrambled control and assessed for Aurora B expression by immunoblot. p85 is shown as the loading control.

Our data suggest a signaling pathway originating from Akt3, leading to WDR12 expression and appropriate chromosome segregation during mitosis. Immunofluorescence was used to establish WDR12 localization during mitosis. During mitosis WDR12 localizes to the mitotic spindle and peppers the chromosomes during telophase (Fig 3A). Considering that chromosome segregation is regulated by Aurora B during the spindle assembly checkpoint (SAC) and that Aurora B localizes to mitotic chromosomes [18] in a similar pattern shown for WDR12, we asked whether WDR12 is associated with Aurora B. Total protein was subjected to immunoprecipitation using antibodies directed against Aurora B and WDR12. As shown in Fig 3B, under endogenous conditions WDR12 associates with Aurora B. To ensure the specificity of the antibodies we used, cells were co-transfected with GFP-tagged Aurora B and Myc-tagged WDR12. We show in Fig 3C, immunoprecipitation using an antibody directed against GFP (Aurora B) results in a co-association with the Myc tag (WDR12), confirming their association. Since inhibition or depletion of Aurora B results in aberrant chromosome segregation [19], we asked whether Akt3 depletion affected Aurora B expression. We found that depletion of Akt3 resulted in a reduction of Aurora B expression (Fig 3C) similar to the Akt3 effect on WDR12 expression [19,20]. Our findings suggest that Akt3 is required for chromosome segregation by controlling the expression of Aurora B and WDR12.

### Mitochondrial-dependent ROS inhibits mitotic fidelity

ROS is a byproduct of mitochondrial electron transport and can cause several detrimental effects during the cell cycle [21]. Research has shown that aberrant accumulation of ROS during mitosis can result in abnormal nuclei and mitotic arrest [22–25]. Indeed, several reports suggest a role for ROS in chromosome missegregation [26,27]. However, to date, exact mechanisms regarding the dysregulation of mitosis by increased ROS have yet to be defined. Our previously published findings show that Akt3 blockade results in decreased mitochondrial homeostasis as shown by decreased oxygen consumption rates, decreased electron transport, mitochondrial fission, and mitochondrial stress [28].

**Fig 4:**
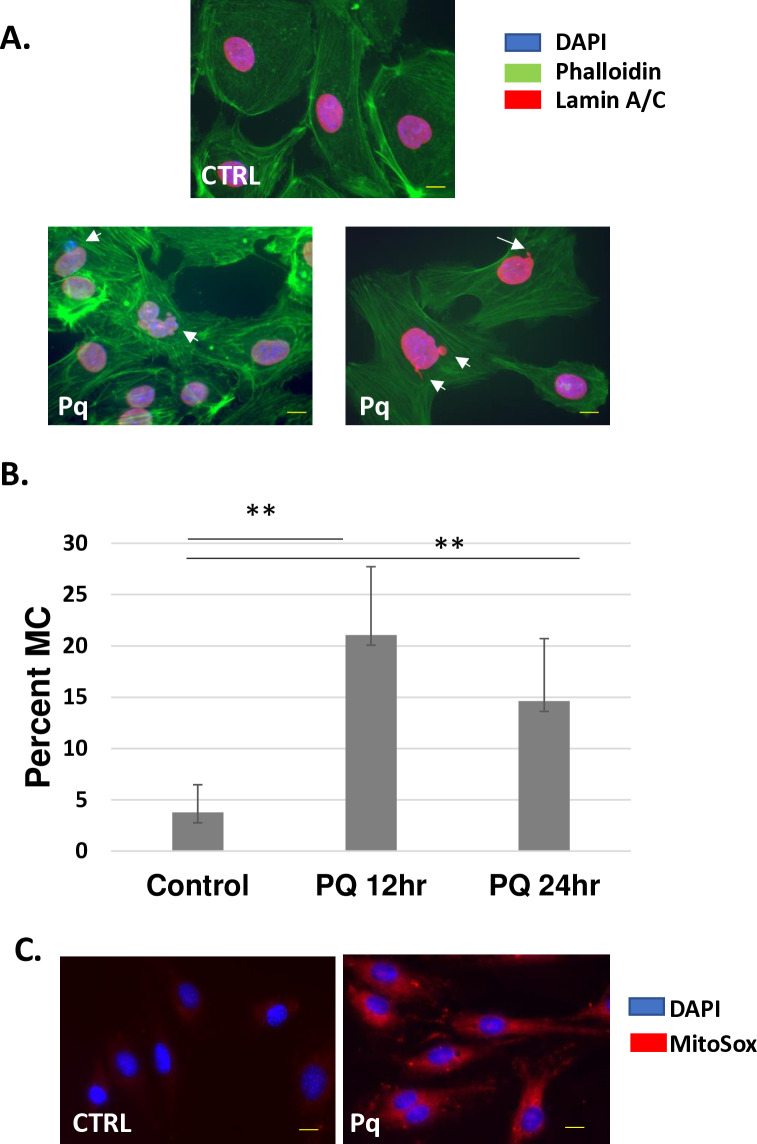
Pharmacological induction of mitochondrial dysfunction results in micronuclei and nuclear blebbing. A) ECs treated with paraquat (1 mM) for 24hr and subjected to immunofluorescence with antibodies directed against lamin A/C, stained with phalloidin and DAPI. Arrows indicate micronuclei and nuclear blebbing. B) Quantitation of micronuclei (MN) in ECs treated with paraquat (1 mM) for 12 or 24hrs. Standard deviations are shown. * p < 0.001, **p < 0,001. C) Mitosox staining of cells treated with paraquat as in A.

To test whether disruption of mitochondrial homeostasis influences mitotic fidelity, we treated ECs with the model oxidant paraquat. As shown in Fig 4A, cells treated with paraquat show increased micronuclei formation and envelope blebbing, similar to that shown for Akt3 depletion. Paraquat treatment causes a 5-fold increase in increased micronuclei formation (Fig 4B) and a 4-fold increase in mitochondrial ROS as measured by MitoSox fluorescence (Fig 4C).

### Antioxidants rescue nuclear morphology

**Fig 5:**
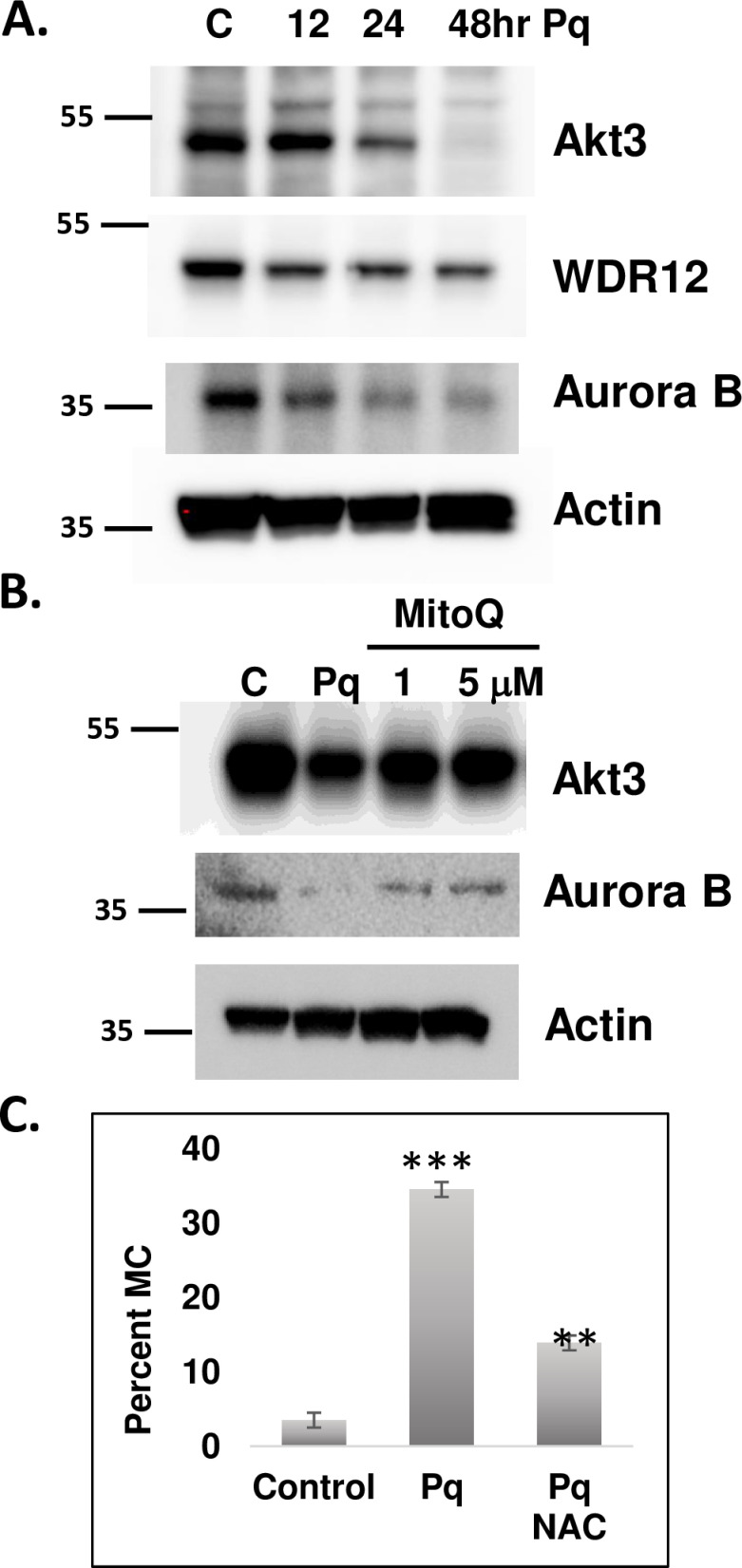
ROS reduces the expression of Akt3, WDR12 and Aurora B leading to micronuclei. A) Paraquat (1 mM) treatment of ECs in a time course analysis and tested for expression of Akt3, WDR12, and Aurora B by immunoblot. Actin is shown as a loading control. B) ECs treated with paraquat plus and minus MitoQ at the concentrations indicated and assessed for Akt3 and aurora B expression by immunoblot. Actin is shown as a loading control. C) Quantitation of percent mitotic catastrophe in ECs treated with paraquat plus and minus the anti-oxidant N-acetyl glucosamine for 24 hours. * p < 0.001, **p < 0,001 Standard deviations are shown.

To test whether paraquat affects the Akt3-WDR12-Aurora B pathway, immunoblots were performed on ECs treated with paraquat in a time-course analysis. As shown in Fig 5A, Akt3 protein is markedly reduced by 24hrs of paraquat treatment. Both WDR12 and Aurora B are also downregulated. Importantly, Mitoquinone mesylate (MitoQ), a mitochondrially directed antioxidant, rescued the effect of paraquat on Akt3 and Aurora B protein levels (Fig 5B). Inhibition of ROS using the antioxidant n-acetyl cysteine (NAC) rescues the effect of paraquat on mitotic fidelity (Fig 5C). Both NAC and MitoQ antioxidants rescue the paraquat effect on mitotic fidelity. Taken together our findings suggest that ROS leads to missegregation of chromosomes via the downregulation of the Akt3/WDR12/Aurora B pathway.

### The Akt3/Aurora B pathway depends on CRM-1

**Fig 6:**
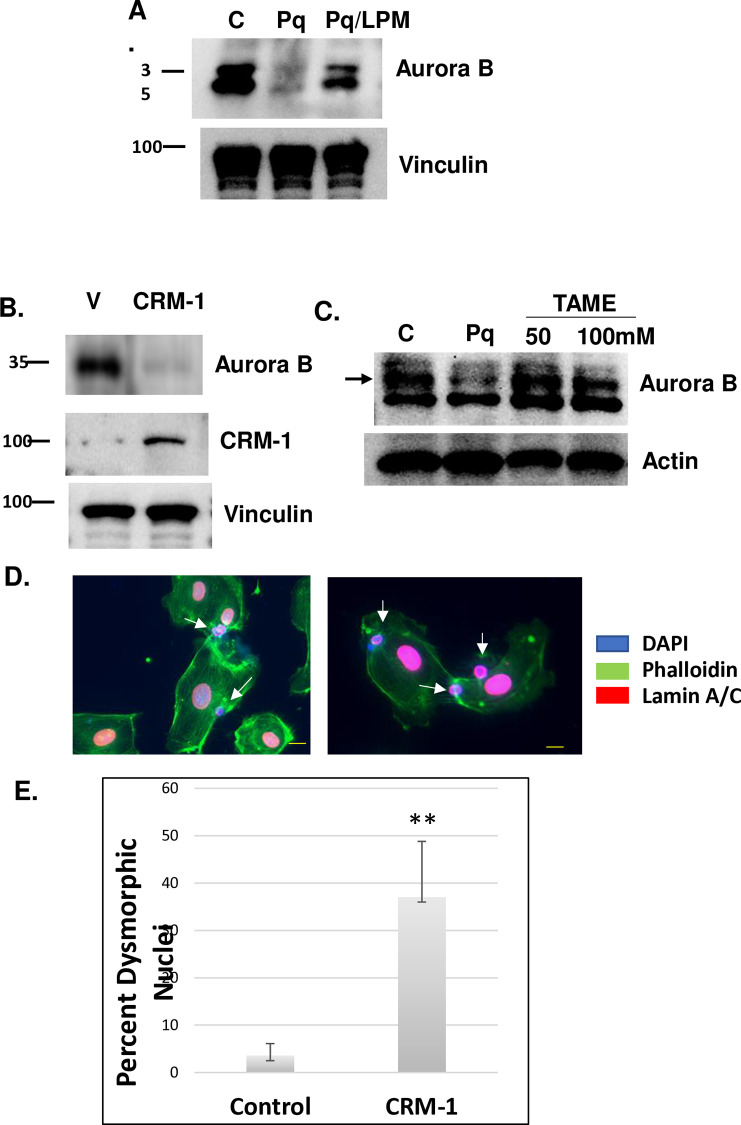
Mitotic catastrophe is dependent on CRM-1 dysregulation. A) EC treated with paraquat (1 mM) for 24hr plus or minus leptomycin B (50 ng/ml) followed by immunoblot for Aurora B. Vinculin is shown as a loading control. B) EC transfected with vector alone or a CRM-1 expression vector assessed for Aurora B expression. Vinculin is shown as a loading control. C) Paraquat treated cells were treated with TAME (50 or 100mM) and assessed for the expression of Aurora B by immunoblot. Actin is shown as an internal control. D) EC subjected to CRM-1 overexpression and assessed for micronuclei using lamin A/C, αtubulin and DAPI staining. E) Quantitation of mitotic catastrophe in CRM-1 overexpressing cells. p < .01.

Our previously published reports show that Akt3 is required for the phosphorylation and destabilization of CRM-1, inhibiting PGC-1 nuclear export. The lack of phosphorylation results in overexpression of CRM-1. To test if the micronuclei formation depended on the misexpression of CRM-1, we treated cells with paraquat plus or minus leptomycin B, an inhibitor of CRM-1 cargo loading. As shown in Fig 6A, leptomycin B treatment rescued the paraquat-dependent downregulation of Aurora B. In addition, CRM-1 overexpression results in a reduction in Aurora B protein expression (Fig 6B). Since Aurora B is a known target of CRM-1 and its nuclear exit results in its degradation by the ubiquitin ligase APC/c [29,30], paraquat treated cells were treated with TAME, a specific APC/c inhibitor. As shown in Fig 6C, inhibition of APC/c rescues the downregulation of Aurora B in response to paraquat treatment. To test whether CRM-1 overexpression resulted in missegregated chromosomes, cells transfected with CRM-1 were assessed for changes in nuclear envelope and micronuclei using antibodies directed against lamin A/C. As shown in Fig 6C and quantitated in Fig 6D, transfected cells have a 7-fold increase in multinucleation and micronuclei formation. These findings suggest that CRM-1 is upstream of Aurora B, and its increased expression under conditions of Akt3 ablation results in mitotic catastrophe.

### Decreased cell proliferation and increased cell death in embryonic Akt3 null mice correlate with smaller hearts and thinner compact layers

**Fig 7:**
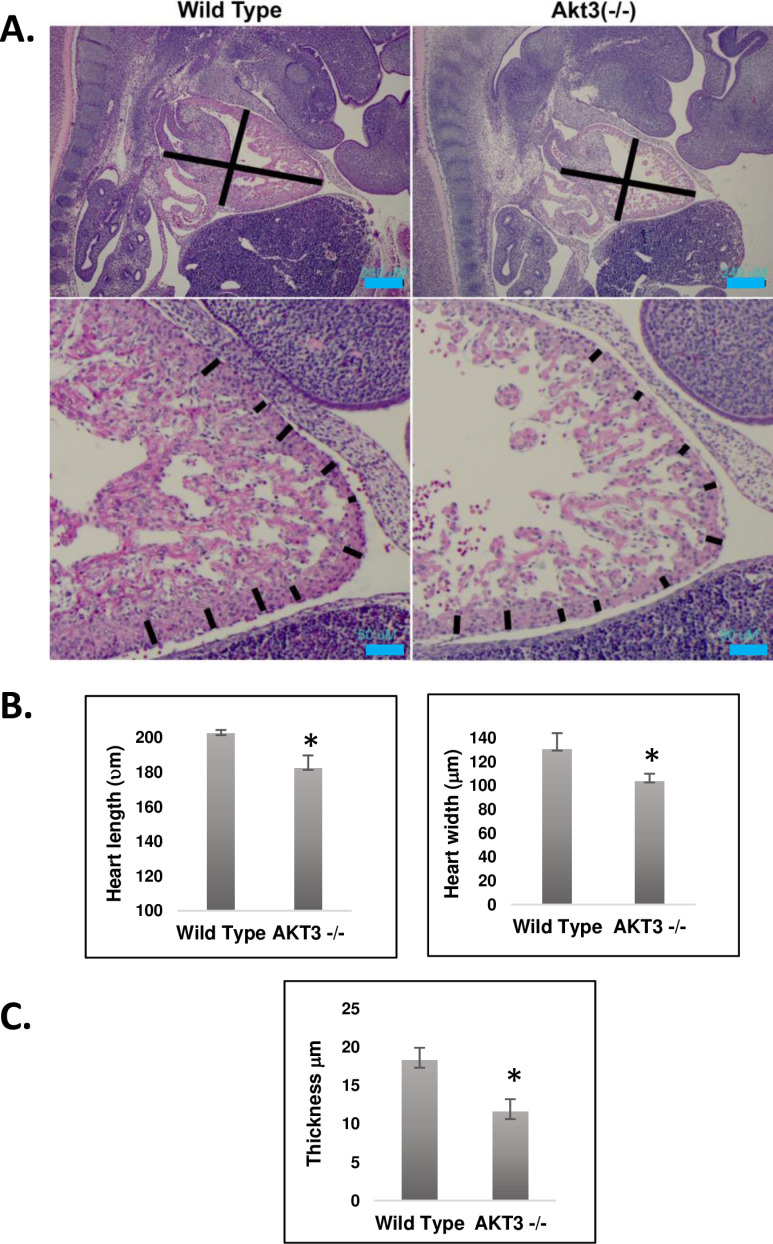
Morphometric analysis of wild type and Akt3 null hearts. A) H&E staining of wild type versus Akt3 hearts. Top panel indicated areas of measurement (length and width). Bottom panel shows areas measured for compact layers. Quantitation is shown in B) length and width and C) thickness of compact layer. n = 2 wild type and n = 4 Akt3 nulls.

Akt3 null and wild type hearts were assessed at E14.5. At this stage, cardiomyocytes are proliferative and the cardiac vasculature is being formed. Akt3 null mouse hearts were significantly smaller in both length (p < 0.05) and width (p < 0.01) (Fig 7B). The thickness of the compact layers of wild-type and null mice were measured at ten different points along the periphery (Fig 7A). Hearts of Akt3 null mice showed a significant decrease in wall thickness (p < 0.01) (Fig 7C). These data are consistent with previous findings but also revealed a previously undescribed defect in the hearts of Akt3 null mice and suggest a reduction in proliferation and/or increased apoptosis.

### Hearts from Akt3 null animals show decreased Aurora B expression and cell proliferation and increased programmed cell death consistent with *in vitro* studies

**Fig 8:**
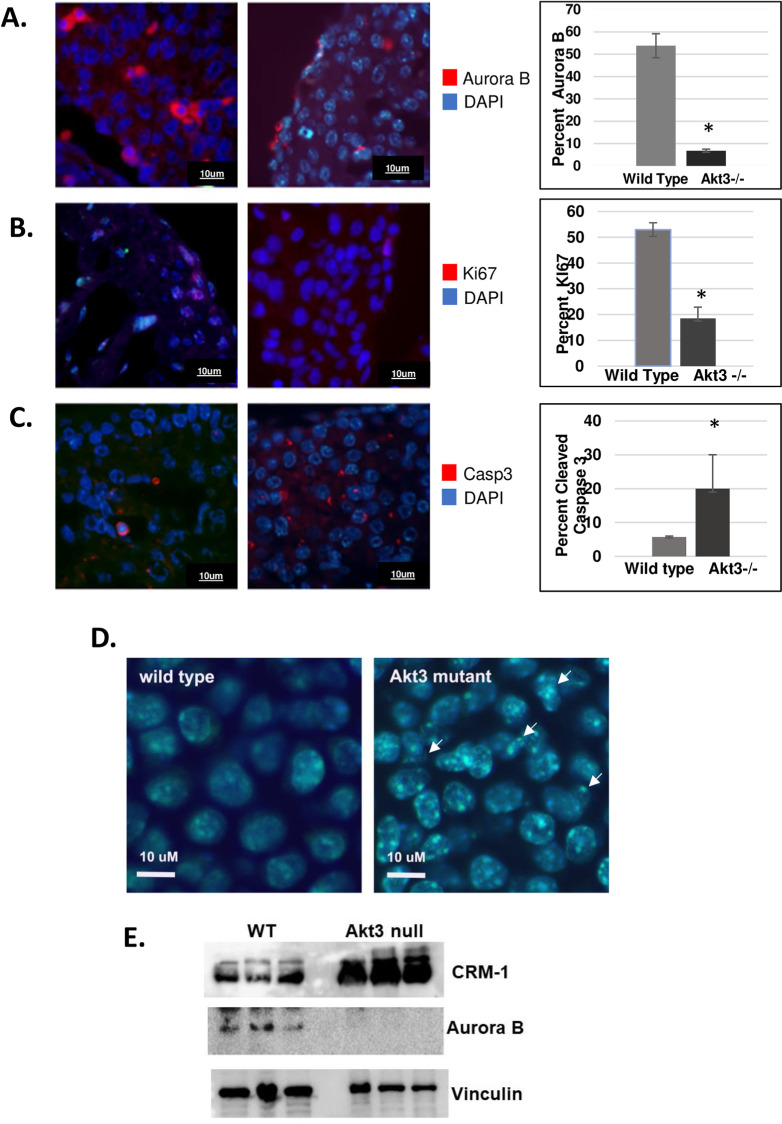
Akt3 null animals have reduced Aurora B expression and proliferation and increased apoptosis. Paraffin sections of Akt3 null hearts were subjected to immunofluorescence using antibodies against A) Aurora B, B) Ki67 and C) cleaved caspase 3. Quantitation is shown next to each figure. D) H&E of wild type and Akt3 null hearts. E) Immunoblot of CRM-1, Aurora B in whole heart lysates of wild type and Akt3 null animals. Vinculin is shown as a loading control.

To determine if our findings linking Akt3 to mitotic regulation are relevant *in vivo* we examined E14.5 hearts from Akt3 null and wild-type mice for markers of proliferation and cell death. Immunohistochemistry studies of Aurora B, Ki67, a general marker of cell proliferation, and activated caspase 3, a marker for programmed cell death (Fig 8) show a 10-fold decrease of Aurora B positive nuclei in Akt3 null mice as compared to wild-type (Fig 8A), phenocopying the *in vitro* results. Only areas of active proliferation were quantitated. Akt3 null hearts also show a 3-fold reduction in Ki67 positive nuclei (Fig 8B) and a 3.5-fold increase in activated caspase 3 as compared to wild-type hearts (Fig 8C). This reduction in Aurora B suggests there is also a mitotic phenotype in Akt3 null heart tissue.

### Akt3 null mice have nuclear abnormalities

Accurate quantitation of chromosome segregation in tissue section is problematic given overlapping cell layers within tissues. However, in a side-by-side comparison of DAPI staining in E14.5 hearts, we noticed aberrant nuclear staining. An example of this staining is shown in Fig 8D. In these stains, we could visualize aberrant nuclear envelope deformation in some nuclei and that the DAPI staining is reduced, suggesting a disruption in nuclear envelope structure similar to that seen *in vitro*. Western blot analysis of total adult heart protein derived from wild-type and Akt3 null animals shows both an increased expression of CRM-1 and a decrease in Aurora B (Fig 8E). Taken together, Akt3 ablation *in vivo* phenocopies several aspects of the *in vitro* analysis: decreased Aurora B expression, increased CRM-1 expression, decreased cell proliferation, increased cell death, and disruption of nuclear morphology.

## Discussion

**Fig 9:**
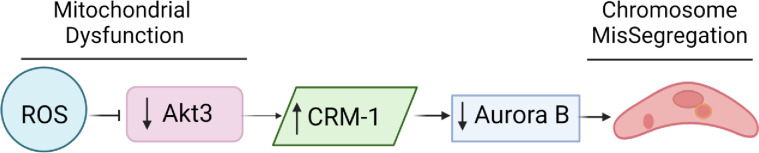
Model of the role of increased ROS in chromosome missegregation. Increased ROS results in a reduction in Akt3 expression. This causes an increased expression of CRM-1 and an APC/c dependent turnover of Aurora B kinase.

Our results support the hypothesis that mitochondrial function is intimately linked to mitosis. Mitochondrial dysfunction leads to the reduction in Aurora B via an Akt3-dependent mechanism that results in micronuclei formation and a nuclear envelopathy. These findings suggest the working model in Fig 9. Increased mitochondrial stress induced by Akt3 depletion or pharmacologically by paraquat results in a reduction in both WDR12 and Aurora B expression driving chromosome missegregation and a nuclear envelopathy. Aurora B is required for appropriate chromosome segregation during the spindle assembly checkpoint; including chromosome microtubule interactions, cohesion, and spindle assembly [31]. Aurora B is also required for nuclear envelope breakdown [32] and cytokinesis [33]. Importantly Aurora B is implicated in nuclear envelope reassembly and is required to inhibit micronuclei formation [34,35].

The exact mechanism by which paraquat treatment downregulates Akt3 remains to be determined. Akt1 is negatively regulated by ROS, with ROS inducing the S-nitrosylation of Akt1 [36,37]. Sequence comparisons between Akt1 and Akt3 show that they share identity at the Akt1 nitrosylation site. Akt3 also contains another potential nitrosylation site not shared by Akt1 [38]. Whether the downregulation of Aurora B is a direct effect of Akt3 depletion is under investigation. Aurora B does not contain potential s-nitrosylation sites as predicted by GPS-SNO [38] but high stringency searches do reveal Akt-specific phosphorylation sites within Aurora B. In addition to the regulation of proliferation and survival, the Akt family of kinases are important regulators of protein expression and stablity. Akt3 is required for the stability of CRM-1 the major nuclear export protein [10] and Aurora B nuclear localization is controlled by CRM-1 [39]. Our findings suggest that in the absence of Akt3, Aurora B is localized to the cytoplasm and turned over by APC/c [40]. Premature Aurora B turnover would result in the missegregation of chromosomes.

We have shown that Akt3 is required for angiogenesis and that overall mitochondrial health; (i.e., respiratory capacity), is a prerequisite for launching angiogenic responses. Rescue of mitochondrial biogenesis by biogenic compounds that increase cAMP can bypass Akt3 depletion and promote angiogenesis [41], suggesting redundancy in pathways capable of restoring mitochondrial homeostasis. These findings confirm the requirement of mitochondrial function for angiogenic responses. However, here we show that Akt3 ablation affects cellular proliferation through the regulation of Aurora B. Cellular proliferation is a process necessary for angiogenesis [42]. During an angiogenic response, stalk cells proliferate in consort with tip cell migration. We propose then that Akt3 depletion causes mitochondrial dysfunction that also affects mitosis in endothelial cells.

WD40 repeat (WDR) proteins are a large family of scaffolding proteins. Here we find that WDR12 interacts with Aurora B and, like Aurora B, is downregulated upon Akt3 depletion. Although WDR12 has historically been considered involved in ribosomal biogenesis in concert with Pes-1 and Bop1 (PeBoW- Pes1-Bop1-WDR12). However, WDR12 is important for cell cycle regulation in yeast, independently of ribosome biogenesis. In yeast, inhibition of WDR12 or its regulatory partners results in aberrant metaphase plates [43]. In *C. elegans*, mutations in the WDR12 protein orthologue result in partial embryonic lethality and slow growth (AceView, NCBI). SNPs within its coding sequence correlate with coronary artery disease [44]*.* Here we show that WDR12 is required for mitosis and that its depletion results in micronuclei formation. WDR12 associates with Aurora B and is downregulated under conditions of Akt3 depletion or paraquat treatment. It has recently been shown that WDR62 associates with Aurora B, is closely associated with the chromosome passenger complex, and mutations in WDR62 are associated with microencephaly [45]. Mutations in Akt3 have been implicated in Megalencephaly-Polymicrogyria-Polydactyly-Hydrocephalus Syndrome (MPPH syndrome) [46,47] where all individuals have a cortical brain malformation called polymicrogyria. That WDR62 mutations are associated with microencephaly is an interesting tie in with mutations in Akt3 in MPPH syndrome. Human mutations that cause constitutive activation of Akt3 result in macrencephaly, while in both humans and mice, null mutations cause microencephaly. Since both WDR62 and Akt3 mutations impinge upon Aurora B, Aurora B dysfunction may be a key regulator of MPPH syndrome.

The exact function of mitochondria in the endothelium is an open question. In endothelial cells, ATP is primarily provided by glycolysis. ATP derived from oxidative phosphorylation is not required to launch an angiogenic response, as inhibition or augmentation of mitochondrial respiration did not affect angiogenic responses *in vitro*. Instead, the ablation of phosphofructokinase-2/fructose-2,6-bisphosphatase 3 (PFKFB3), a key glycolytic enzyme, can halt angiogenesis altogether [48]. Our studies point to an important function for mitochondrial homeostasis during angiogenesis. Disruption of mitochondrial function results in mitotic effects, affecting endothelial cell proliferation. A nuclear envelopathy also has important consequences for the biology of the cell, causing global changes in gene expression. The nuclear position of the chromatin can affect the transcription of genes and can result in numerous human diseases including congenital muscular dystrophy, myopathy, and cardiomyopathy [49].

Overexpression of Akt family members in mouse models activates myocyte hypertrophy and the subsequent overall increase in heart size [26,27]. Histological investigation of Akt1 null mice demonstrates its importance for heart development and function since Akt1-deficiency leads to heart defects and decreased cell proliferation [17]. Similarly, overexpression of Akt3 in hearts promotes cardiac growth and mediates cardioprotective effects by reduction of programmed cell death in the short-term, although sustained activation of Akt3 causes contractile dysfunction and progressive maladaptive hypertrophy [9]. The exact role of Akt3 in the heart is less clear, with loss of function studies giving variable results. Both Akt1 and Akt3 null mice are viable, but Akt1-/-; Akt3 -/ + mice die shortly after birth with various anatomical heart defects as well as non-proportional hypotrophy of the thymus, skin, and brain [50]. Interestingly, other studies of Akt3 null mice saw variable results, with, for example, Easton et al. reporting a slight increase in the weight of Akt3 null hearts [51] but most others reporting smaller hearts. Akt3 is also differentially expressed in the hearts of diabetic model mice, further implicating it as an important player in cardiac development. Diabetic mice have increased incidences of congenital heart defects [52]. These variable results may be due to differences in genetic background, but further studies are needed. In humans, Akt3 has also been identified as a downstream target of microRNA-29C, a known biomarker for congenital heart defects [53]. In adult hearts, Akt3 has been implicated as a key mediator of the anti-apoptotic effects of miRNA-145 during myocardial infarction [53].

Mitochondrial biogenesis and quality control are essential to human health. Dysfunction at any single step has implications in multiple pathologies. Indeed, mtDNA mutations are implicated in cancer pathogenesis [54]. Impaired mitochondrial homeostasis is implicated in multiple neurological diseases, including Alzheimer’s disease [55]. Defects in mitochondrial fission/fusion dynamics have been demonstrated to cause Dominant Optic Atrophy, and improper mitochondrial degradation has been implicated in the development of Parkinson’s disease [56]. As the role of the mitochondria in the vasculature is much less clear, studies focusing on this organelle within the vascular endothelium will undoubtedly contribute to the understanding of multiple cardiovascular pathologies, including those implicating oxidative stress, such as hypertension and diabetic vascular dysfunction [57]. Our data demonstrate the requirement of Akt3 in mitochondrial homeostasis, the regulation of chromosome segregation through its effect on Aurora B kinase, and for appropriate nuclear envelope formation. Indeed, data in this study suggests the Akt3 pathway is required for communication between the mitochondria and nucleus. Future studies will investigate the role of Akt3 as a nodal kinase in the regulation of mitochondrial homeostasis in human disease.

## Supporting information

S1 File Supplemental data 3. (XLSX)

S2 File Supplemental data files. (PDF)

S3 File Supplemental data 1. (PDF)
