# Correction: Akt3 links mitochondrial function to the regulation of Aurora B and mitotic fidelity

**DOI:** 10.1371/journal.pone.0343145

**Published:** 2026-02-17

**Authors:** The PLOS One Staff

In Fig 1B, the labels in the x axis are incorrect. Please see the correct Fig 1 here.

The publisher apologizes for the errors.

**Fig 1:**
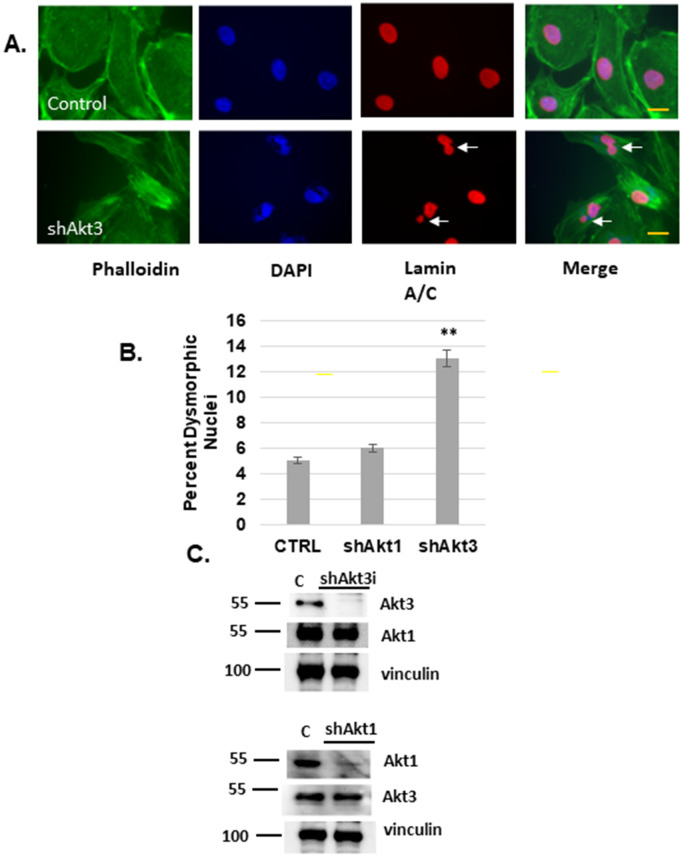
Akt3 depletion results in micronuclei formation. **A)** ECs were subjected to Akt3 depletion or scrambled control. Fixed cells were assessed for nuclear structure using immunofluorescence with antibodies directed against Lamin A/C and actin stained with phalloidin. DAPI is used as a nuclear stain. Arrows point to multinuclei and blebbing. 60X images are shown **B)** Quantitation of micronuclei (MN) under conditions of scrambled control, or Akt1 or Akt3 depletion. Standard deviation and p values are shown. **C)** Confirmation of Akt1 and Akt3 knockdown by immunoblot analysis. The PI3 kinase p85 subunit is shown as a loading control.
