# Supplementary figures and images for: Akt3 links mitochondrial function to the regulation of Aurora B and mitotic fidelity
Source: PLoS One. 2025 Mar 6;20(3):e0315751. doi: 10.1371/journal.pone.0315751 (PMC11884723; doi:10.1371/journal.pone.0315751)

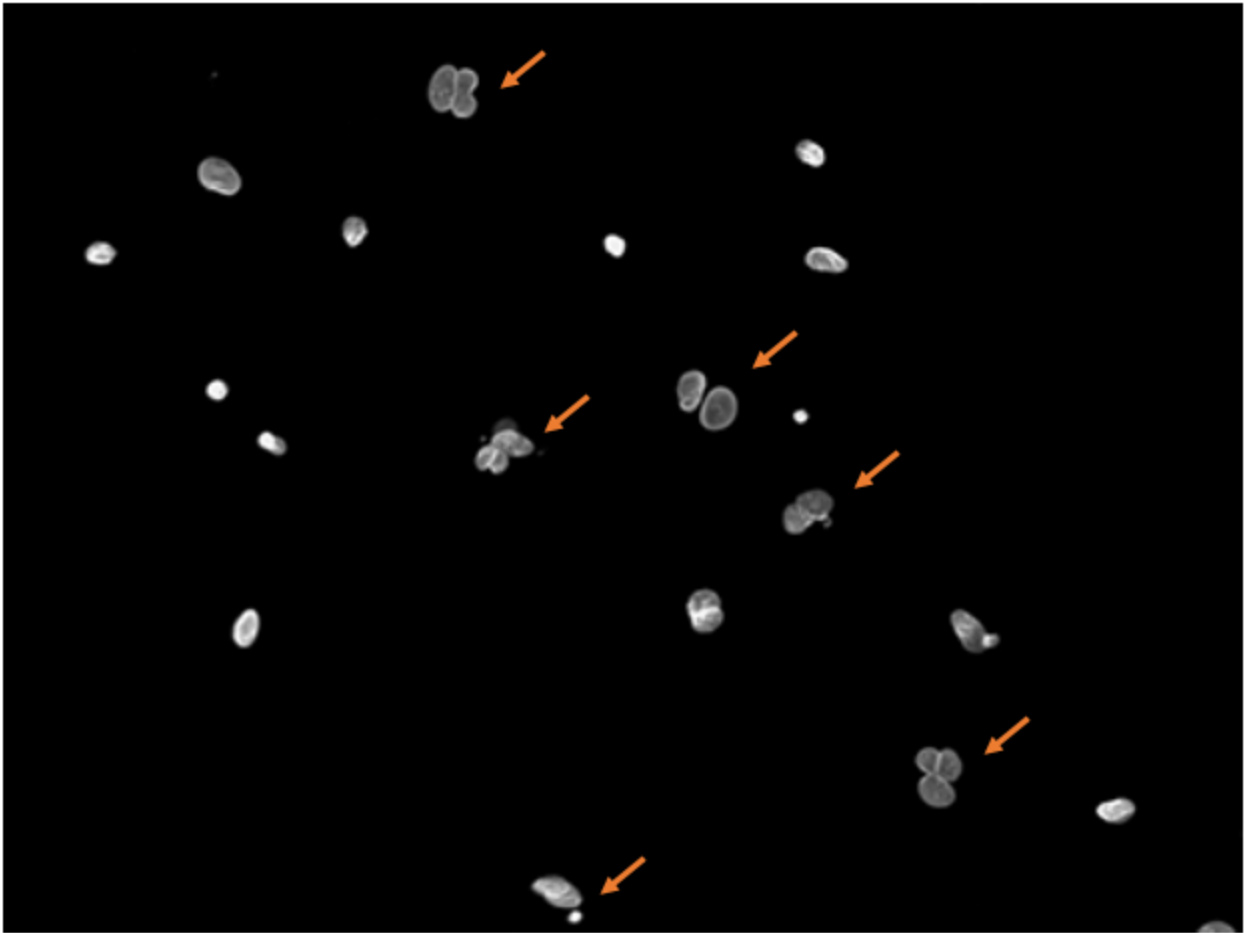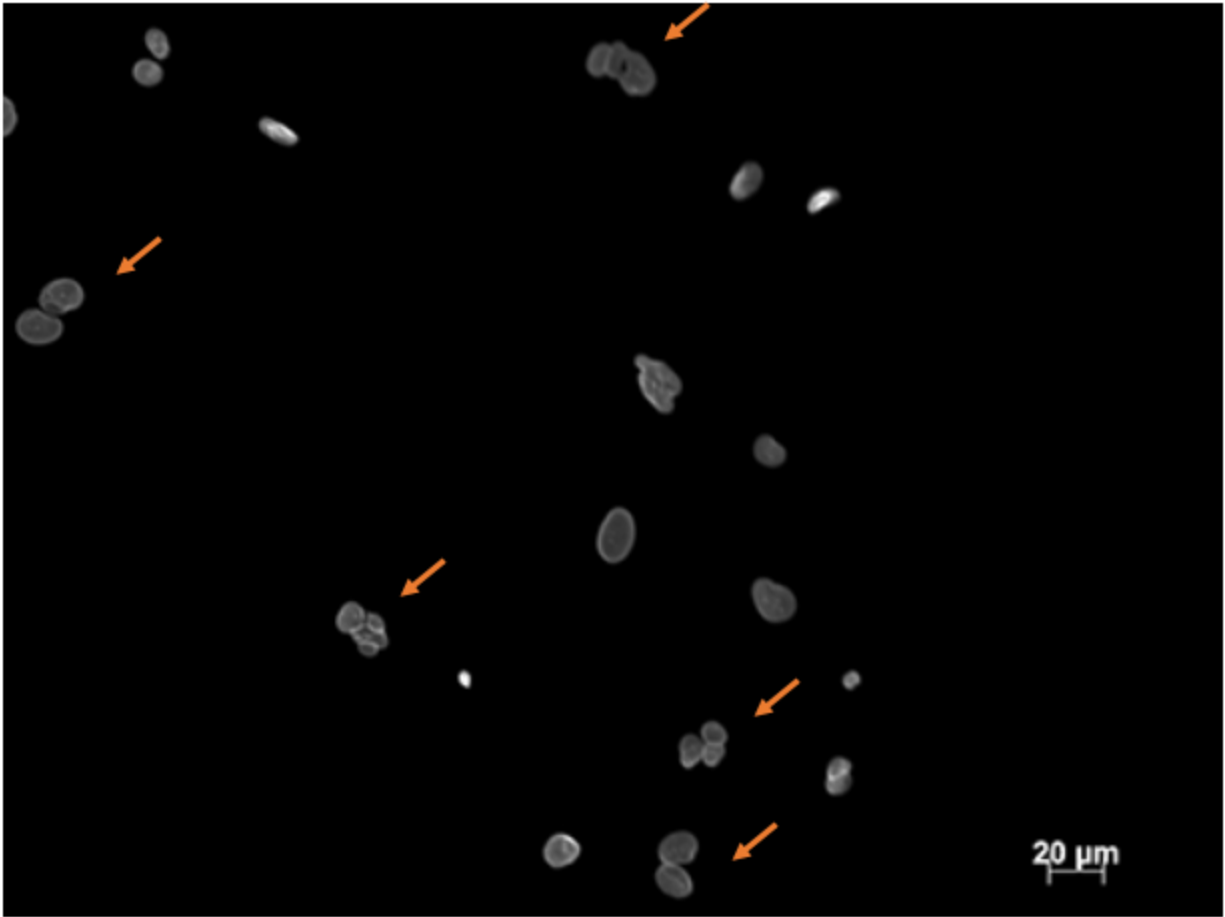

Supplement: S2 File — (PDF) [file pone.0315751.s002.pdf]
