# Supplementary material for: Akt3 links mitochondrial function to the regulation of Aurora B and mitotic fidelity
Source: PLoS One. 2025 Mar 6;20(3):e0315751. doi: 10.1371/journal.pone.0315751 (PMC11884723; doi:10.1371/journal.pone.0315751)

Fig. 1

shAkt3 Akt3 IB

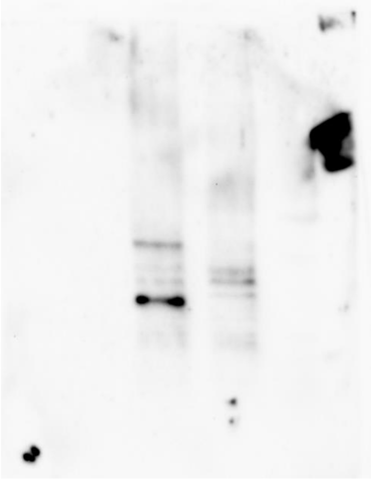

Vinculin IB

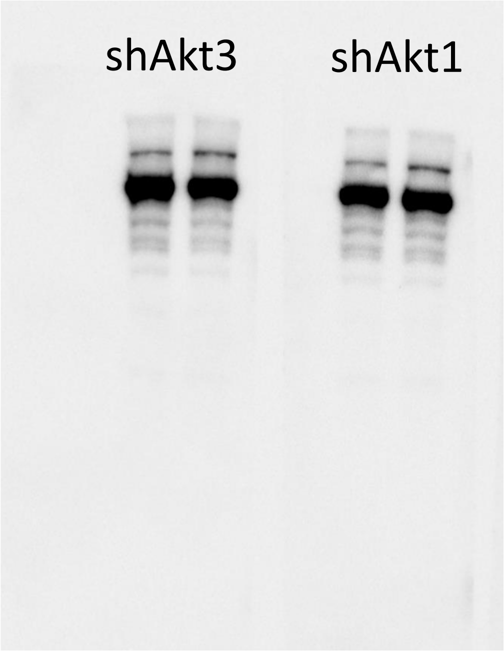

shAkt3 Akt1 IB

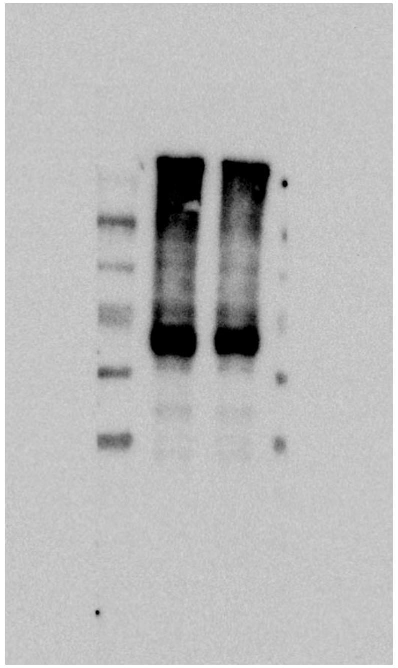

Fig. 1

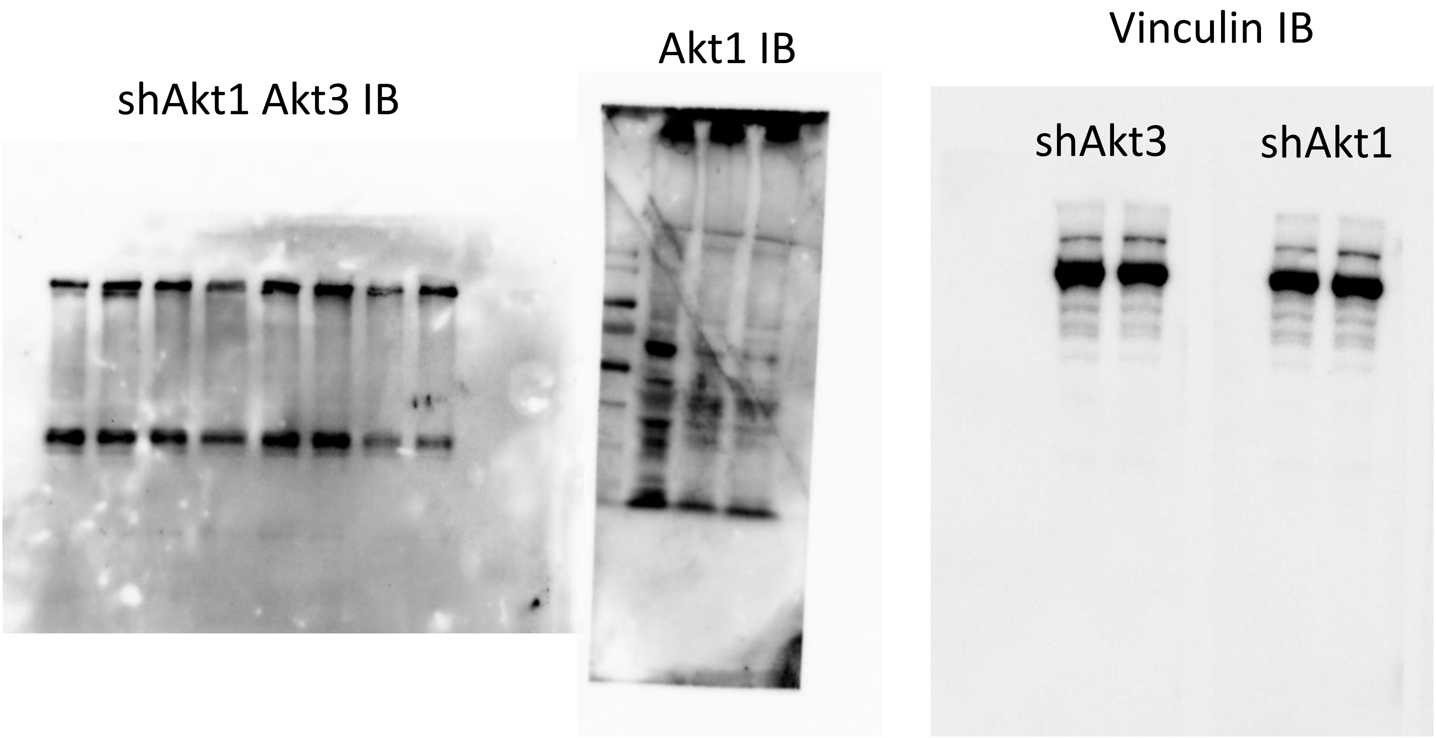

Fig. 2

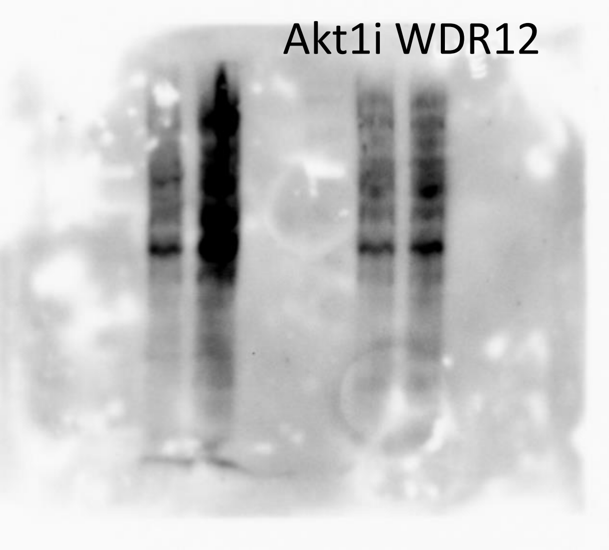

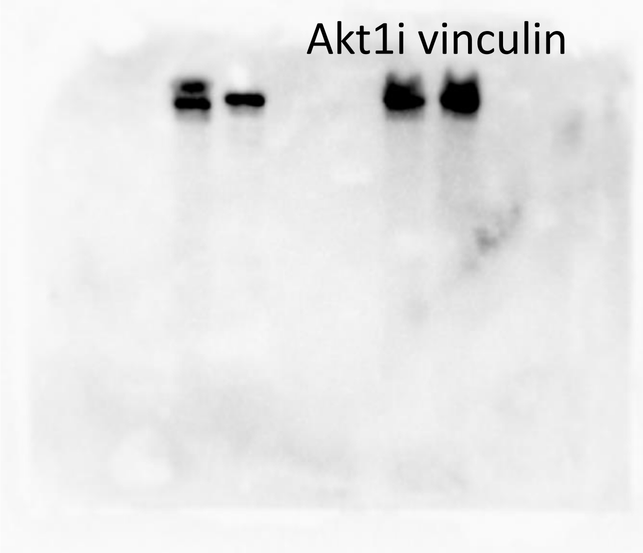

Akt3i WDR12

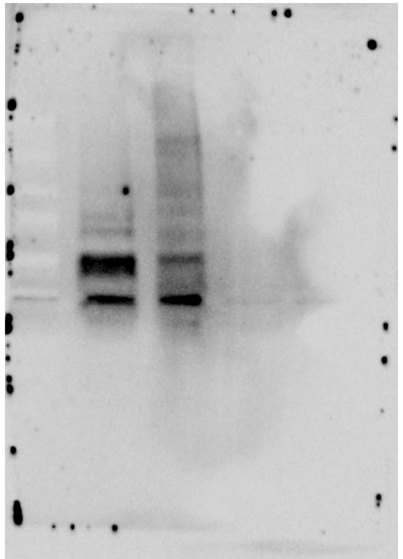

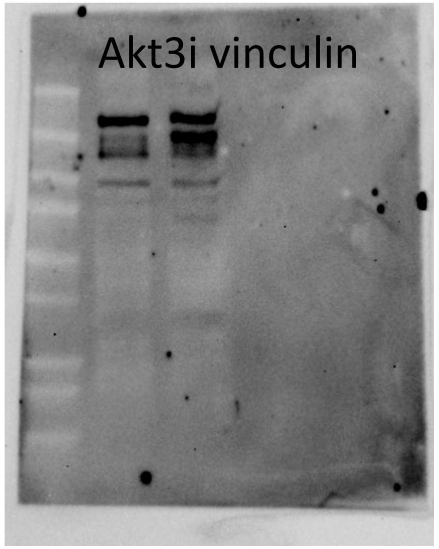

Fig. 2

WDR12 over

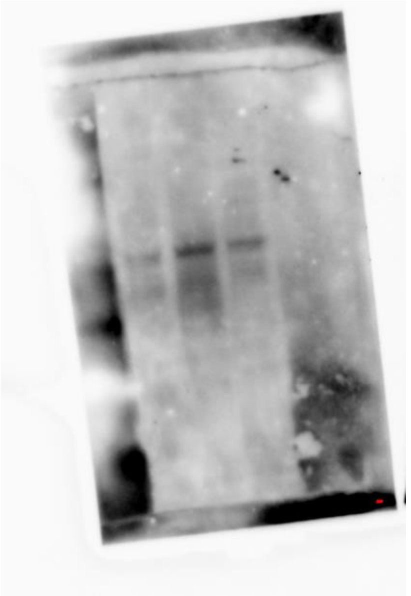

WDR12 over

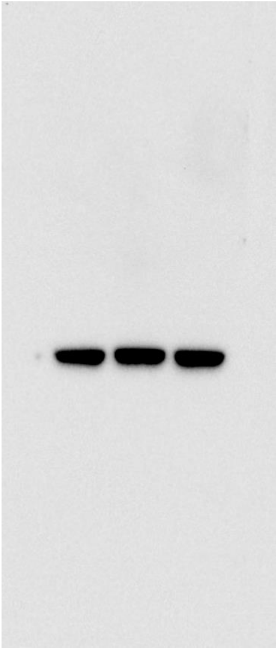

actin

Fig3

IP WDR12 IB Aurora

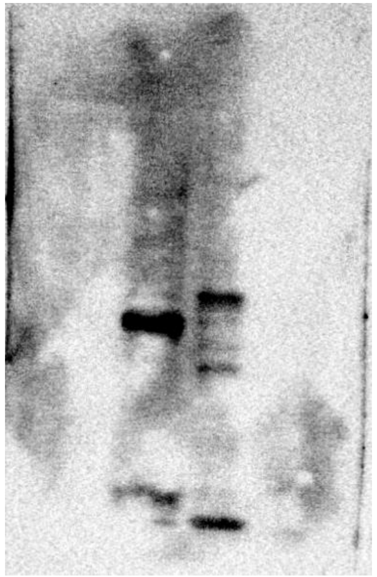

IP GFP IB Myc

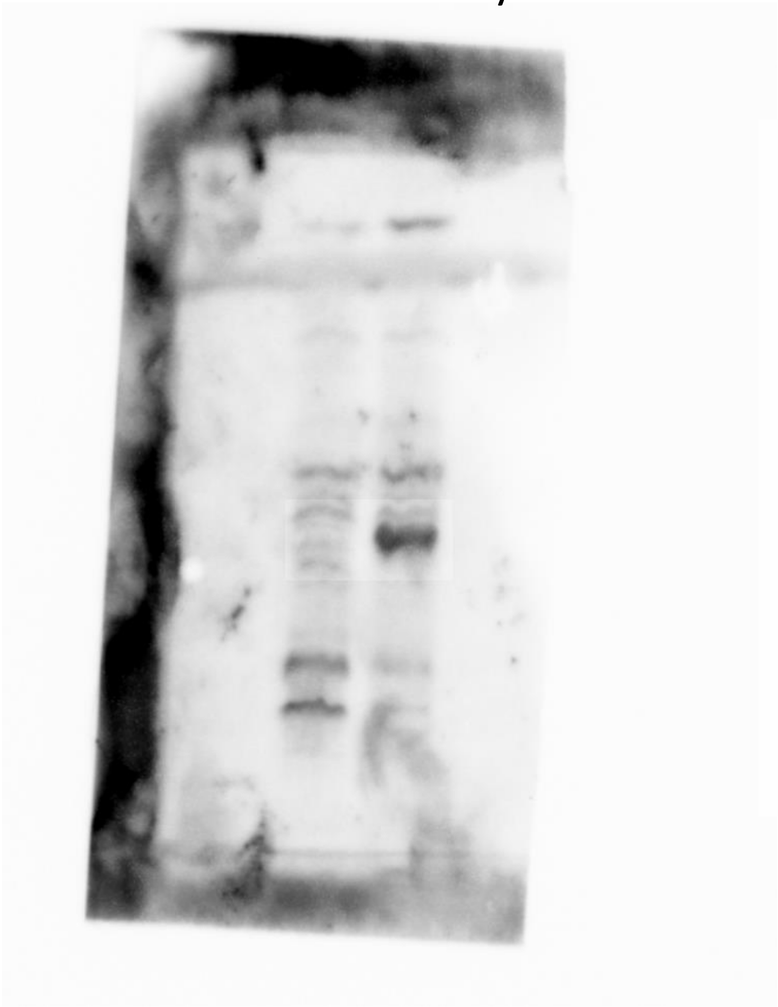

IP Aurora IB WDR12

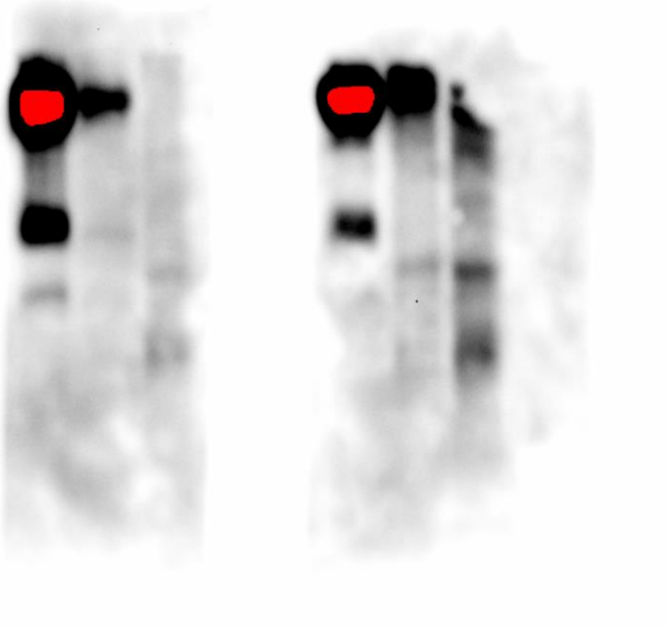

Fig3

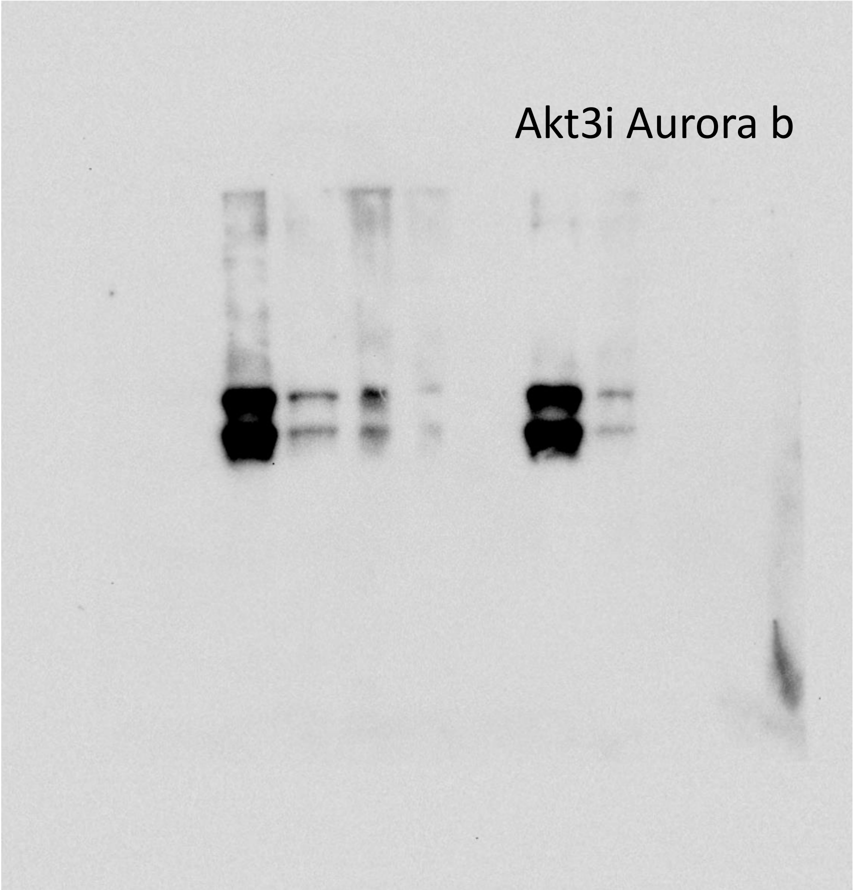

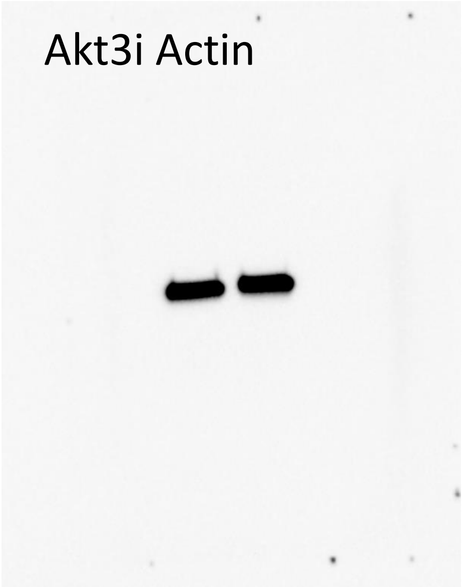

Figure 5

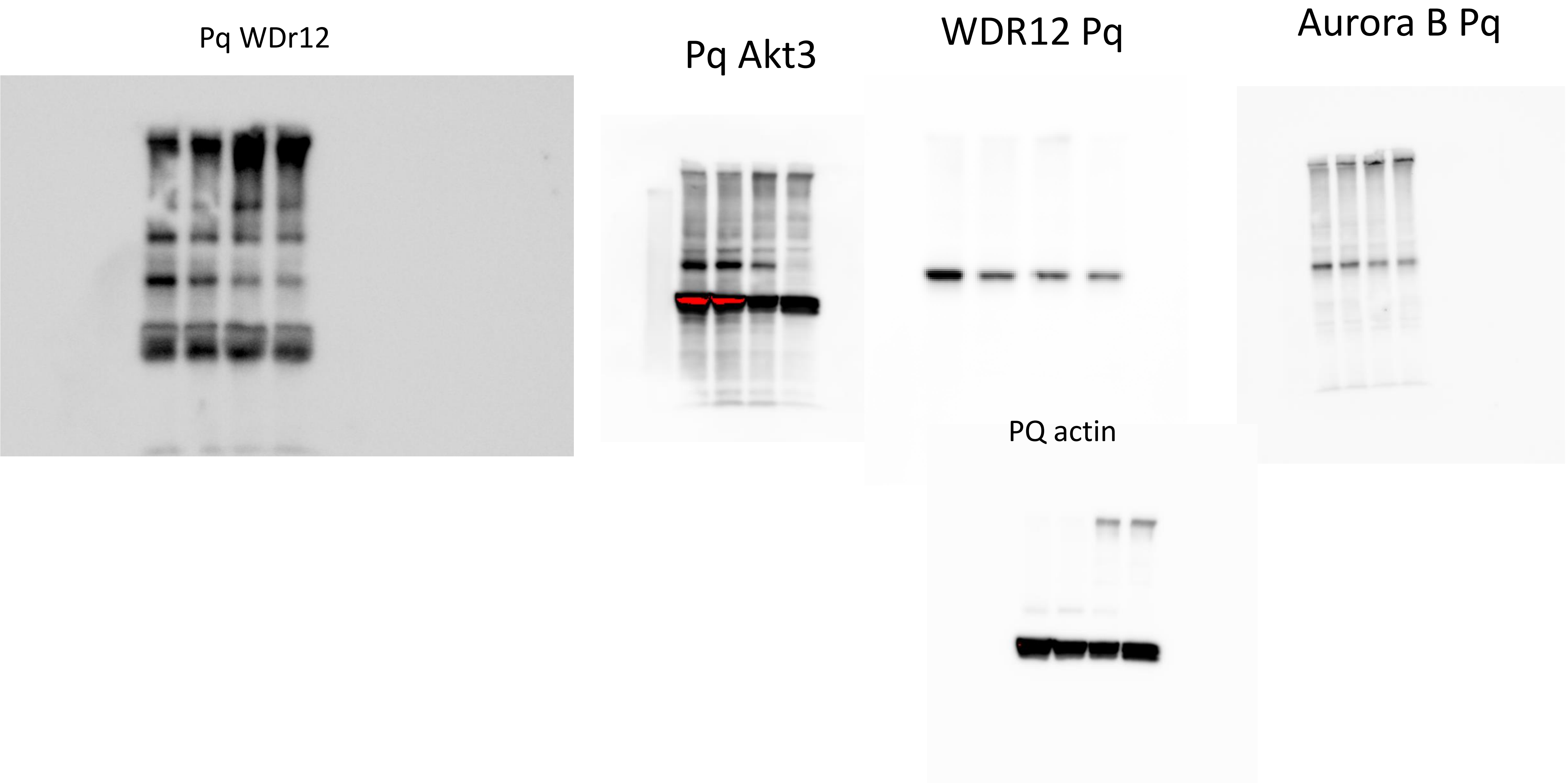

Figure 5

Akt3 pq mitoq

Aurora B Pq

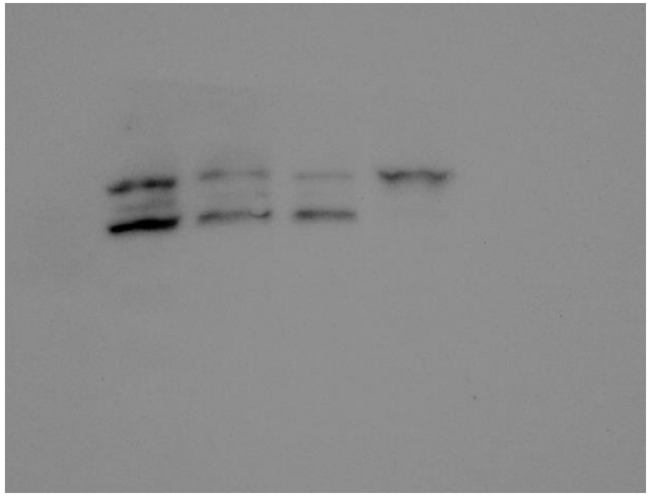

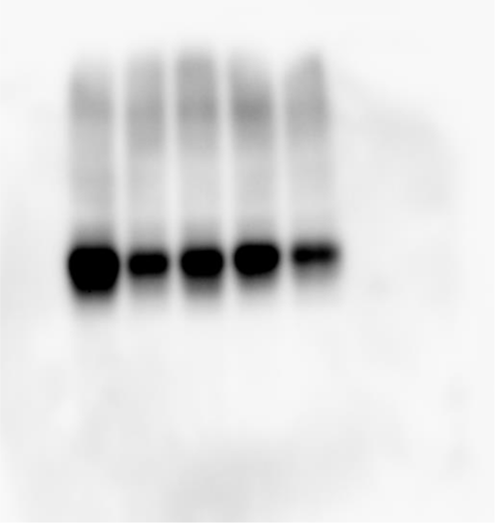

Actin

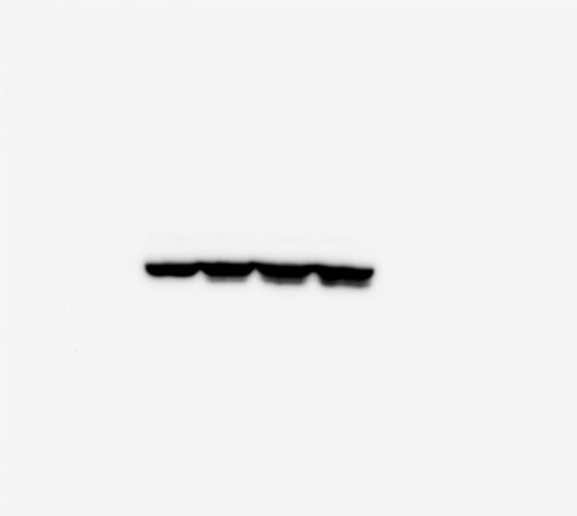

Figure 6

C pq pqlpm pq aurora B

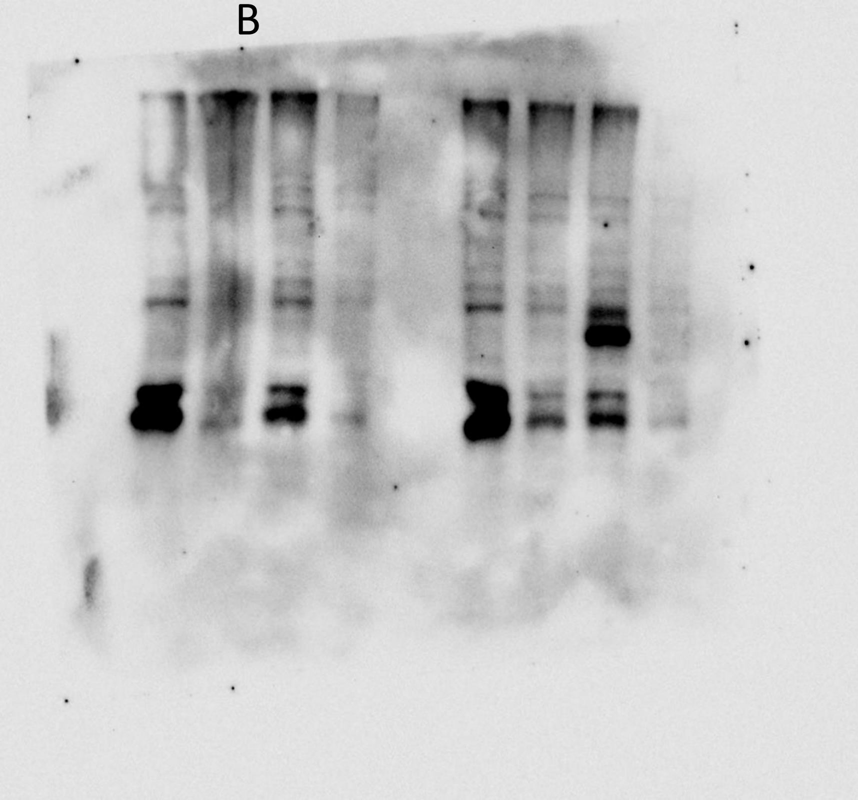

Vinculin

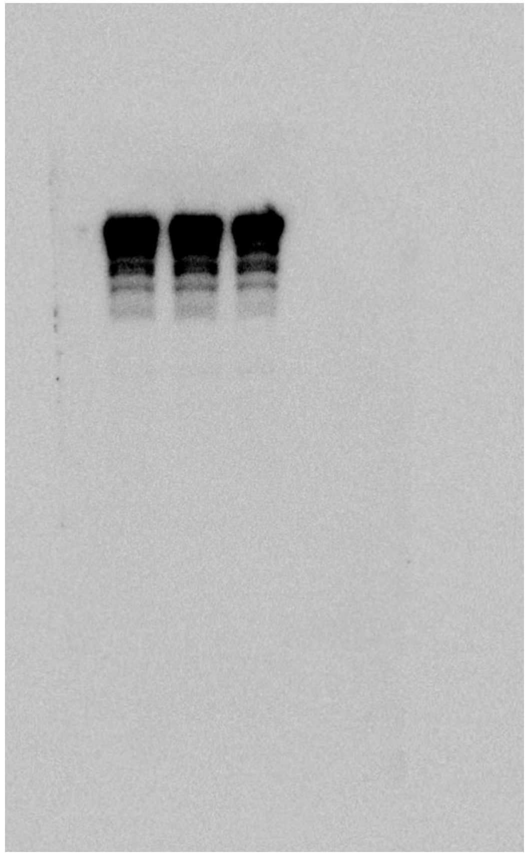

CRM-1 over Aurora B

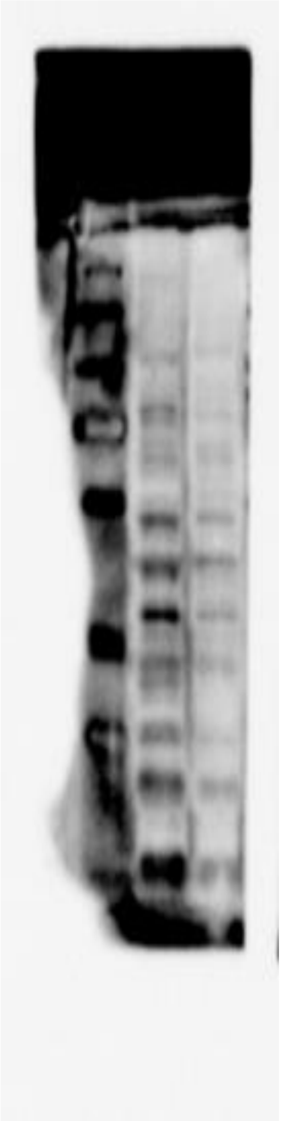

CRM-1 over Vinculin

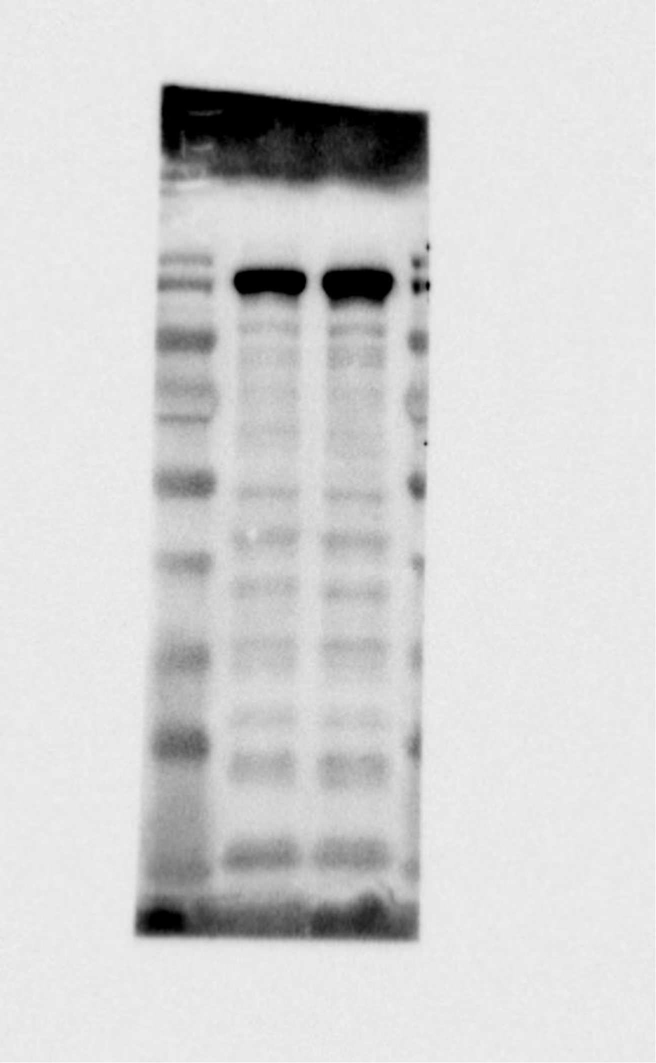

Figure 6

CRM over

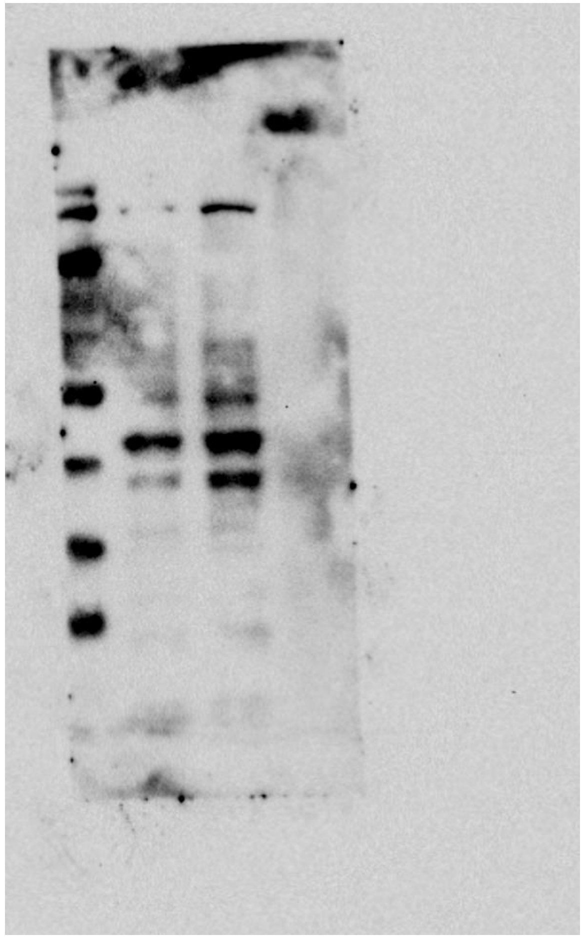

Aurora pq Tame MG132

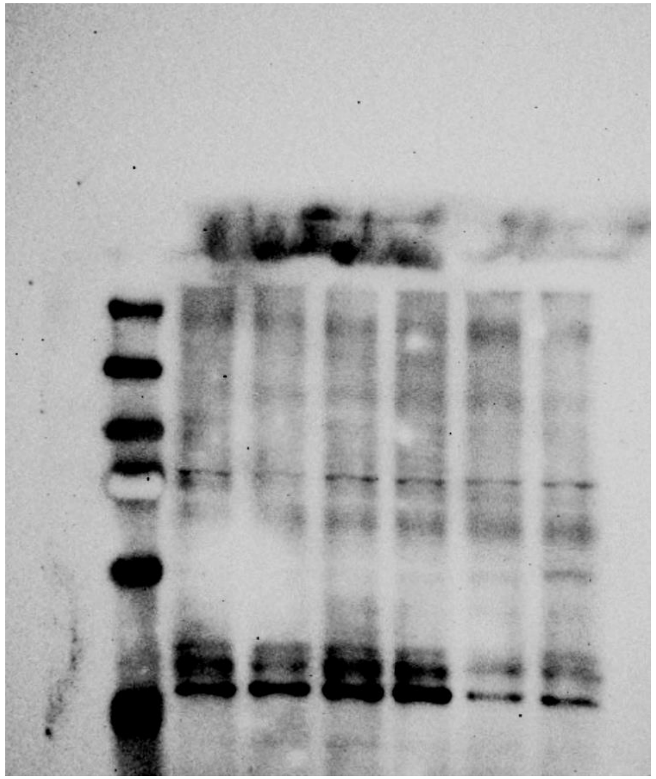

Actin pq Tame

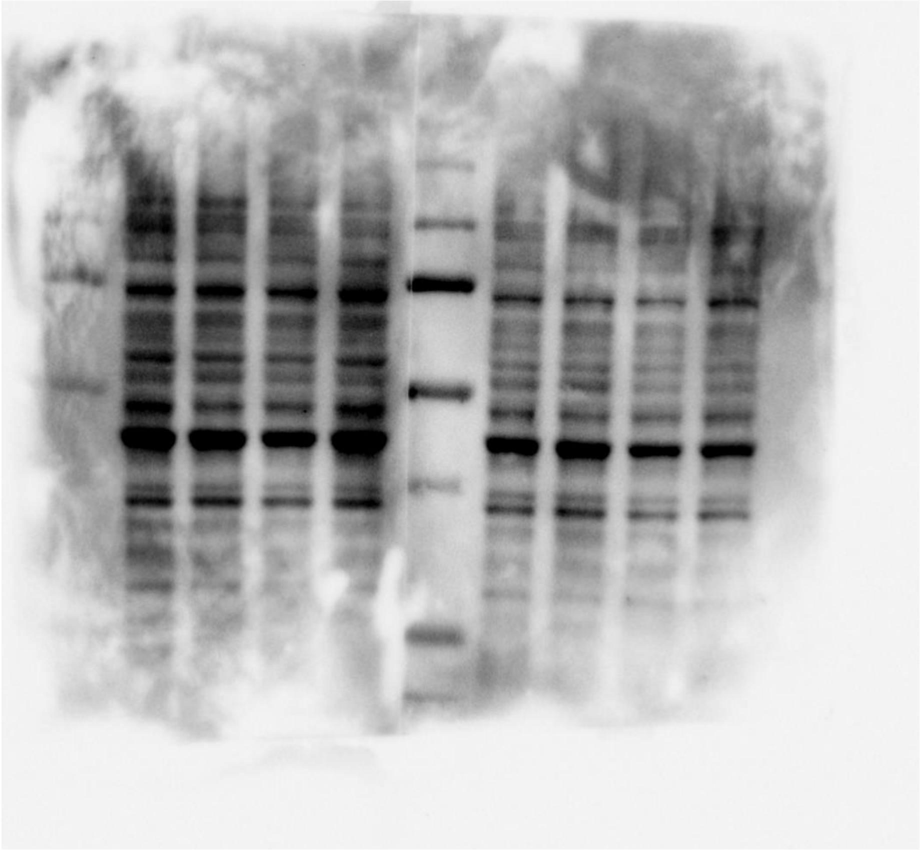

Figure 8

Heart CRM-1

Heart Aurora B

Heart vinculin

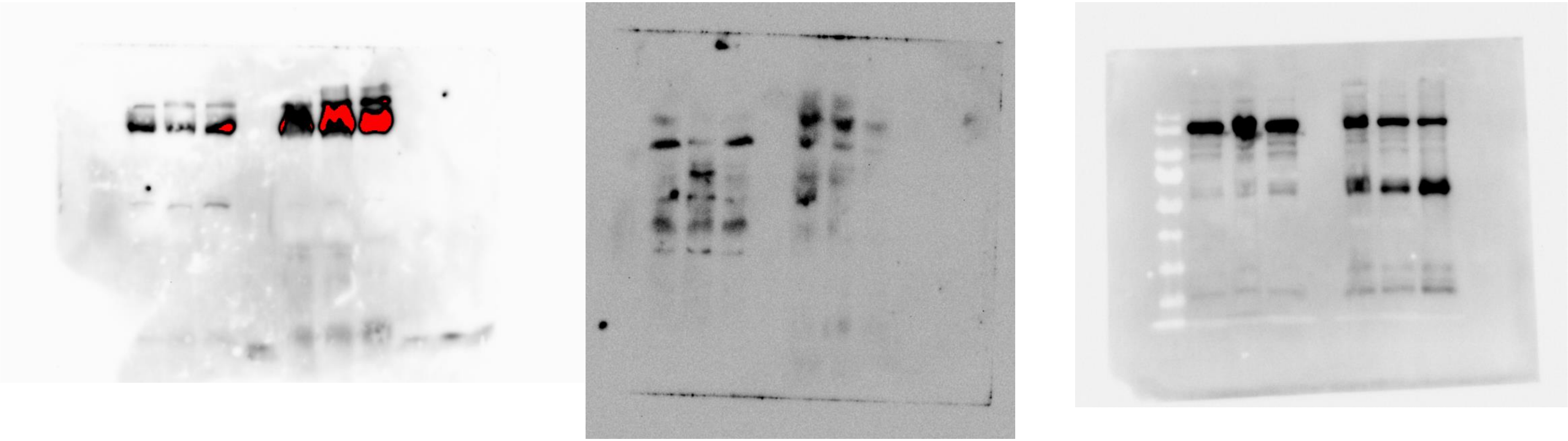

Supplement: S3 File — (PDF) [file pone.0315751.s003.pdf]
